# Kidins220 sets the threshold for survival of neural stem cells and progenitors to sustain adult neurogenesis

**DOI:** 10.1038/s41419-023-05995-7

**Published:** 2023-08-04

**Authors:** Ana del Puerto, Coral Lopez-Fonseca, Ana Simón-García, Beatriz Martí-Prado, Ana L. Barrios-Muñoz, Julia Pose-Utrilla, Celia López-Menéndez, Berta Alcover-Sanchez, Fabrizia Cesca, Giampietro Schiavo, Miguel R. Campanero, Isabel Fariñas, Teresa Iglesias, Eva Porlan

**Affiliations:** 1 Instituto de Investigaciones Biomédicas “Alberto Sols”, Consejo Superior de Investigaciones Científicas-Universidad Autónoma de Madrid (CSIC-UAM), C/ Arturo Duperier, 4, 28029 Madrid, Spain; 2 Centro de Investigación Biomédica en Red de Enfermedades Neurodegenerativas (CIBERNED), Instituto de Salud Carlos III, Av, Monforte de Lemos, 3-5. Pabellón 11. Planta 0, 28029 Madrid, Spain; 3 Departamento de Biología Molecular, Universidad Autónoma de Madrid, C/ Francisco Tomás y Valiente, 7, Ciudad Universitaria de Cantoblanco, 28049 Madrid, Spain; 4 Centro de Biología Molecular “Severo Ochoa” (CSIC-UAM), C/ Nicolás Cabrera, 1, 28049 Madrid, Spain; 5 Instituto Universitario de Biología Molecular – UAM, C/ Nicolás Cabrera, 1, 28049 Madrid, Spain; 6 Departmento de Biología Celular, Biología Funcional y Antropología Física, Universidad de Valencia, C/ Dr. Moliner, 50, 46100 Burjassot, Spain; 7 Department of Life Sciences, University of Trieste, via L. Giorgieri, 5, 34127 Trieste, Italy; 8 Department of Neuromuscular Disorders, UCL Institute of Neurology, University College London, London, WC1N 3BG UK; 9 UK Dementia Research Institute, University College London, London, WC1E 6BT UK; 10 Centro de Investigación Biomédica en Red en Enfermedades Cardiovasculares (CIBERCV), Instituto de Salud Carlos III, Av. Monforte de Lemos, 3-5. Pabellón 11. Planta 0, 28029 Madrid, Spain; 11 Instituto de Investigación Sanitaria del Hospital Universitario La Paz (IdiPAZ), Instituto de Salud Carlos III, Av. Monforte de Lemos, 3-5. Pabellón 11. Planta 0, 28029 Madrid, Spain; 12 Present Address: Departamento de Biotecnología, Instituto Nacional de Investigación y Tecnología Agraria y Alimentaria (INIA-CSIC), Autovía A6, Km 7,5, 28040 Madrid, Spain; 13 Present Address: Centro de Biología Molecular “Severo Ochoa” (CSIC-UAM), C/ Nicolás Cabrera, 1, 28049 Madrid, Spain

**Keywords:** Adult neurogenesis, Neural stem cells

## Abstract

In the adult mammalian brain, neural stem cells (NSCs) located in highly restricted niches sustain the generation of new neurons that integrate into existing circuits. A reduction in adult neurogenesis is linked to ageing and neurodegeneration, whereas dysregulation of proliferation and survival of NSCs have been hypothesized to be at the origin of glioma. Thus, unravelling the molecular underpinnings of the regulated activation that NSCs must undergo to proliferate and generate new progeny is of considerable relevance. Current research has identified cues promoting or restraining NSCs activation. Yet, whether NSCs depend on external signals to survive or if intrinsic factors establish a threshold for sustaining their viability remains elusive, even if this knowledge could involve potential for devising novel therapeutic strategies. Kidins220 (Kinase D-interacting substrate of 220 kDa) is an essential effector of crucial pathways for neuronal survival and differentiation. It is dramatically altered in cancer and in neurological and neurodegenerative disorders, emerging as a regulatory molecule with important functions in human disease. Herein, we discover severe neurogenic deficits and hippocampal-based spatial memory defects accompanied by increased neuroblast death and high loss of newly formed neurons in Kidins220 deficient mice. Mechanistically, we demonstrate that Kidins220-dependent activation of AKT in response to EGF restraints GSK3 activity preventing NSCs apoptosis. We also show that NSCs with Kidins220 can survive with lower concentrations of EGF than the ones lacking this molecule. Hence, Kidins220 levels set a molecular threshold for survival in response to mitogens, allowing adult NSCs growth and expansion. Our study identifies Kidins220 as a key player for sensing the availability of growth factors to sustain adult neurogenesis, uncovering a molecular link that may help paving the way towards neurorepair.

## Introduction

In the mammalian adult brain, two specialized microenvironments contribute to the maintenance of neural stem cell (NSC) pools to sustain continuous neurogenesis throughout life. In these niches, NSCs and their own cellular progeny coexist with supporting cells, a specialized extracellular matrix and vasculature. The mouse subependymal zone (SEZ) comprises a layer of cells juxtaposed to the ventricular wall lined by ependymal cells, amidst which residing NSCs extend a primary cilium to contact the cerebrospinal fluid and sense the milieu. In the SEZ, relatively quiescent NSCs (B cells) give rise to fast-dividing intermediate progenitor cells (C cells). Arising from these progenitors are neuroblasts (type A cells) that migrate into the olfactory bulb (OB), to differentiate into several types of interneurons, that contribute to refine the processing of olfactory information [1]. Similarly, in the subgranular zone (SGZ) of the hippocampal dentate gyrus (DG), NSCs termed radial glia-like (RGL)-type 1 cells [2] give rise to intermediate progenitors (type 2a and 2b cells), which generate new neurons that migrate into the DG cell layer where they integrate into hippocampal circuits [3].

The generation of new neurons as an ongoing dynamic process echoes in adulthood the mechanisms driving neurogenesis during neurodevelopment. The number of NSCs and newly generated neurons can be modulated at all phases of the process by restraining or promoting proliferation, differentiation, migration, survival, maturation, and integration of newborn neurons into the existing circuitry [3, 4].

Stress, aging, and neurodegeneration are notable negative regulators of adult neurogenesis involved in memory loss, mood alterations and additional behavioral changes, as revealed by studies in rodents (reviewed in [5]). In contrast, dysregulation of proliferation and survival of NSCs have been hypothesized to be at the origin of glioma (reviewed in [6, 7]). Thus, dissecting the molecular basis of the regulated transition that NSCs must undergo from quiescence to activation to generate new progeny is of considerable relevance since it bestows potential for devising novel stem cell-based therapeutic strategies. At the molecular level, in NSCs this transition is governed by numerous factors, either inherent to them or to the niche [3, 4], and even systemic to the entire organism [8]. Although short and long-distance cues have shown effects in the activation of adult NSCs, little is known about how their survival, serving as a requisite for proliferation, is regulated. Whether NSCs are dependent on external signals to survive or whether intrinsic factors establish a threshold for sustaining the viability of these cell populations is unclear [9].

Kidins220 (Kinase D-interacting substrate of 220 kDa) [10], also known as ARMS (Ankyrin repeat-rich membrane spanning) [11], is an essential gene whose roles keep emerging. It functions as a signaling scaffold at the plasma membrane as an effector of several signaling pathways. Kidins220 acts downstream of neurotrophin receptors and interacts with diverse signaling pathways to promote neuronal survival, differentiation, and synaptic activity [12, 13], and acts as a regulator of nervous system development [14, 15]. KIDINS220 expression is altered in cancer and in neurological and neurodegenerative disorders, including cerebral ischemia, Alzheimer’s disease, Huntington´s disease and idiopathic normal pressure hydrocephalus [16–23]. Additionally, rare novel missense and loss-of-function variants in *KIDINS220* gene are associated with schizophrenia [24, 25] and, more recently, SINO (spastic paraplegia, intellectual disability, nystagmus, and obesity) syndrome and ventriculomegaly [26–28], altogether identifying KIDINS220 as a multifaceted player with important functions in human disease.

Herein, we identify Kidins220 as a novel intrinsic regulator of NSCs in adult neurogenic niches. We report that Kidins220 decreased expression provokes severe neurogenic deficits, increases neuroblast death and loss of newborn neurons in the SGZ and impairs hippocampal-based spatial memory . Mechanistically, we demonstrate that Kidins220-dependent activation of AKT in response to epidermal growth factor (EGF) restraints glycogen synthase kinase-3 (GSK3) activity preventing NSCs apoptosis. We also show that Kidins220 loss limits the capacity of EGFRs to respond to their ligands, activate and enhance downstream survival cascades in NSCs. Thus, this study identifies Kidins220 as a key player for sensing the availability of growth factors to sustain adult neurogenesis, uncovering a molecular mechanism by which Kidins220 confers NSCs responsiveness to mitogens, setting a molecular threshold for NSC survival.

## Results

### Kidins220 expression in cellular populations of the adult subependymal and subgranular zones

**Fig. 1:**
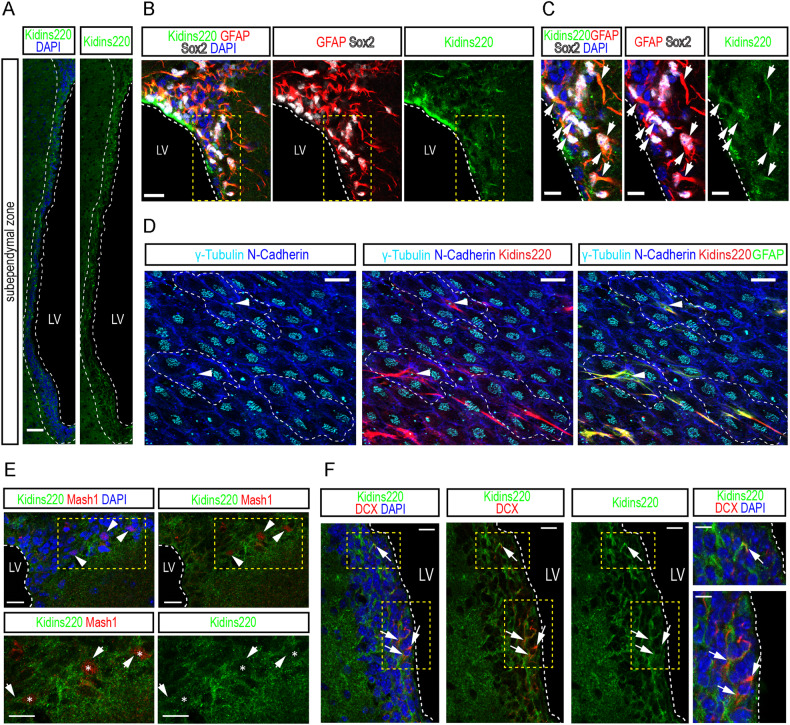
Kidins220 expression in cellular populations of the adult subependymal zone. **A** Confocal micrographs of the staining for Kidins220 (green) in the subependymal zone (SEZ, white dashed lines) of wild-type mice. Nuclei are stained with DAPI (blue). Scale bar 50 μm. **B,** **C** Confocal micrographs of the staining for GFAP (red), Sox2 (gray) and Kidins220 (green) in the SEZ. Triple-positive cells are shown (white arrowheads). Nuclei are stained with DAPI (blue). Scale bar 20 μm. White dashed lines indicate the boundaries of the lateral ventricle (LV), and yellow dashed lines delimit insets magnified in **C** (scale bar, 10 μm). **D** Representative confocal micrographs of lateral ventricle wholemount *en face* preparations stained for N-cadherin (blue), γ-tubulin (cyan), Kidins220 (red) and GFAP (green) to reveal the characteristic pinwheel structure (white dashed lines) where SEZ stem cells reside (white arrowheads). Scale bar 20 μm. **E** Confocal micrographs of the staining for Mash1 (red) and Kidins220 (green) in the SEZ. Double-positive cells are shown (asterisks, white arrowheads). Nuclei are stained with DAPI (blue). Scale bar 20 μm, and 10 μm (insets). **F**, Confocal micrographs of the staining for DCX (red) and Kidins220 (green) in the SEZ. Double-positive cells are shown (white arrowheads). Nuclei are stained with DAPI (blue). Scale bar 20 μm, and 10 μm (insets).

First, we studied Kidins220 expression in the SEZ of adult mice using specific antibodies [10, 20, 23], and observed it is abundant in this neurogenic niche (Fig. 1A). To examine Kidins220 expression by NSCs, we combined Kidins220 antibodies with sex-determining region Y-box 2 (Sox2) transcription factor (expressed by B1 cells and by progenitors [29] and glial fibrillar acidic protein (GFAP; expressed by B1 stem cells [3]. In the SEZ, Kidins220 immunostaining was particularly bright in GFAP^+^-Sox2^+^ astrocytes localized in the vicinity or in direct contact with the walls of the lateral ventricle (Fig. 1B, C). SEZ GFAP^+^ NSCs span amongst multiciliated ependymal cells, exposing a small portion of their membrane with a primary cilium to the ventricle lumen, in so-called ‘pinwheel’ structures. Ciliary (both motile and primary) basal bodies can be labelled with antibodies against γ-tubulin [30]. As shown (Fig. 1D), anti-Kidins220 antibodies stained very strongly NSCs, i.e., GFAP^+^-cells with a primary cilium (γ-tubulin^+^), located within a rosette of multiciliated ependymal cells, labeled with N-Cadherin [30, 31]. To assess the expression pattern of Kidins220 in activated (a) NSCs/C cells and in neuroblasts, we performed costainings for the transcription factor Mash1 and the microtubule-binding protein DCX (Fig. 1E, F, respectively), finding that it is indeed expressed by these populations, as well as in differentiated neurons, as shown by costainings with NeuN (Fig. S1).

**Fig. 2:**
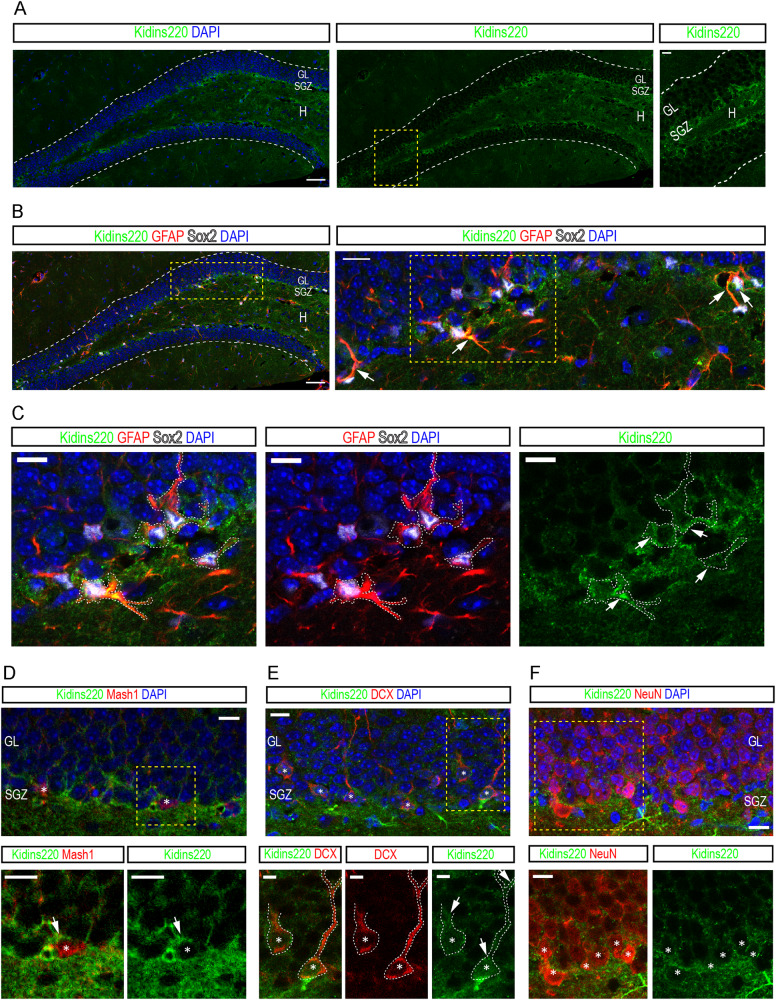
Kidins220 expression in cellular populations of the adult subgranular zone. **A** Confocal micrographs of the staining for Kidins220 (green) in the dentate gyrus (DG, white dashed lines) of wild-type mice. Nuclei are stained with DAPI (blue). Scale bars: 50 μm and 10 μm (inset). SGZ, subgranular zone; GL, granule cell layer; H, hilus. **B** Confocal micrographs of the staining for GFAP (red), Sox2 (gray) and Kidins220 (green) in the DG of wild-type mice. Nuclei are stained with DAPI (blue). Scale bars: 50 μm, 25 μm (inset). White arrows point at triple-positive cells (inset). **C**, Detail images of the staining of Kidins220 (green) in GFAP (red)-Sox2 (gray)-double-positive cells (white dashed lines, white arrows). Scale bar: 10 μm. **D** Confocal micrographs of the staining for Mash1 (red) and Kidins220 (green) in the SGZ. Double-positive cells are shown (asterisks, white arrows). Nuclei are stained with DAPI (blue). Scale bars 10 μm. **E** Confocal micrographs of the staining for DCX (red) and Kidins220 (green) in the SGZ. Double-positive cells are shown (white dashed lines, white arrows). Nuclei are stained with DAPI (blue). Scale bars: 10 μm, and 5 μm (insets). **F** Confocal micrographs of the staining for NeuN (red) and Kidins220 (green) in the SGZ and GL. Double-positive cells are shown (white asterisks). Nuclei are stained with DAPI (blue). Scale bars: 20 μm, and 10 μm (insets). Yellow dashed lines delimit insets magnified in **A**, **B**, **D**, **E** and **F**.

We next inspected the SGZ. As for the SEZ, we found that Kidins220 expression was especially abundant in the *hilus* and the layer of cells comprising the SGZ (Fig. 2A). Immunodetection of Sox2 and GFAP to label RGL-type 1 cells combined with Kidins220 antibodies showed that Kidins220 was also observed in NSCs (Fig. 2B, C). Kidins220 was also found in Mash1^+^ aNSCs/progenitors, in DCX^+^ neuroblasts and in NeuN^+^ granule neurons (Fig. 2D–F), being thus expressed in all relevant populations for adult neurogenesis in both niches.

### Kidins220 deletion in adult neural stem cells

**Fig. 3:**
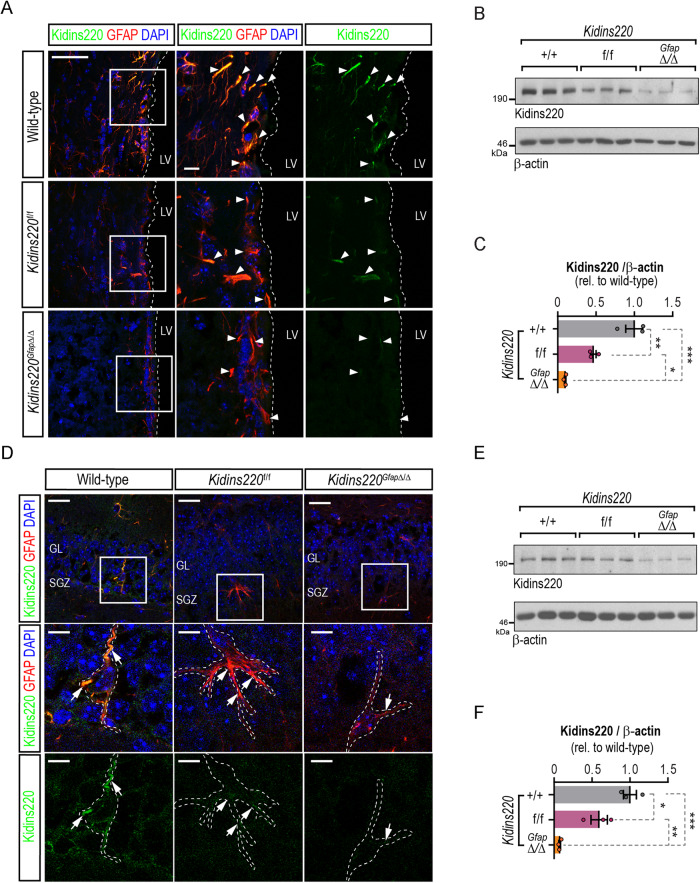
Kidins220 deletion in adult neural stem cells from adult neurogenic niches. **A** Confocal micrographs of the staining for GFAP (red) and Kidins220 (green) in the SEZ of wild-type, *Kidins220*^*f/f*^ and *Kidins220*^*GfapΔ/Δ*^ mice. Nuclei are stained with DAPI (blue). Scale, 50 μm, and 10 μm (insets). LV, lateral ventricle (delimited by white dashed lines). White arrowheads point at GFAP positive cells. **B** Representative Kidins220 and ß-actin (loading control) immunoblots of SEZ lysates from wild-type, *Kidins220*^*f/f*^ and *Kidins220*^*GfapΔ/Δ*^ mice. Each lane represents extracts from one mouse. **C**, Kidins220 levels represented in arbitrary units after normalization with ß-actin in wild-type, *Kidins220*^*f/f*^ and *Kidins220*^*GfapΔ/Δ*^ mice. Data represent mean ± s.e.m.; each data point represents an individual mouse (*N* = 3, for each condition). *P < 0.05, **P < 0.01, ***P < 0.001, by one-way ANOVA followed by Tukey’s *post-hoc* test. **D** Confocal micrographs of the staining for GFAP (red) and Kidins220 (green) in the DG of wild-type, *Kidins220*^*f/f*^ and *Kidins220*^*GfapΔ/Δ*^ mice. Nuclei are stained with DAPI (blue). Scale bars: 25 μm, and 10 μm (insets). SGZ, subgranular zone: GL, granule cell layer; White arrowheads point at GFAP^+^ cells (white dashed lines). **E** Representative Kidins220 and ß-actin (loading control) immunoblots of hippocampal lysates from wild-type, *Kidins220*^*f/f*^ and *Kidins220*^*GfapΔ/Δ*^ mice. Each lane represents extracts from one mouse. **F** Kidins220 levels represented in arbitrary units after normalization with ß-actin in hippocampal lysates in wild-type, *Kidins220*^*f/f*^ and *Kidins220*^*GfapΔ/Δ*^ mice (*N* = 3, each). Data represent mean ± s.e.m.; each data point represents an individual mouse. *P < 0.05, **P < 0.01, ***P < 0.001, by one-way ANOVA followed by Tukey’s *post-hoc* test.

*Kidins220*-floxed (*Kidins220*^*f/f*^) mice have been described previously as a hypomorphic model of Kidins220 and display reduced levels of Kidins220 when compared to control mice in different tissues [23]. The SEZ in *Kidins220*^*f/f*^ animals also showed Kidins220 protein downregulation, detected in fixed tissue and homogenates (Fig. 3A–C). To obtain a complete deletion of Kidins220 in adult NSCs, we crossed *Kidins220*^*f/f*^ animals with mice expressing the Cre recombinase under the mouse *Gfap* promoter [32], to produce *Kidins220*^*Gfap*Δ/Δ^ mice. Immunoblot in cultured primary astrocytes from wild-type, *Kidins220*^*f/f*^ and *Kidins220*^*GfapΔ/Δ*^ mice showed complete Kidins220 ablation (Fig. S2). Kidins220 was undetectable in GFAP^+^ cells from *Kidins220*^*GfapΔ/Δ*^ mice in the SEZ (Fig. 3A), and barely detectable in protein lysates form this location (Fig. 3B–C). Likewise, in the SGZ we found decreased expression in GFAP^+^ cells in *Kidins220*^*f/f*^ tissue, and below the detection threshold in GFAP^+^ cells from *Kidins220*^*GfapΔ/Δ*^ mice (Fig. 3D), which was confirmed in hippocampal tissue extracts by immunoblot (Fig. 3E, F).

### Neurogenic defects in the hippocampus of Kidins220 deficient mice

**Fig. 4:**
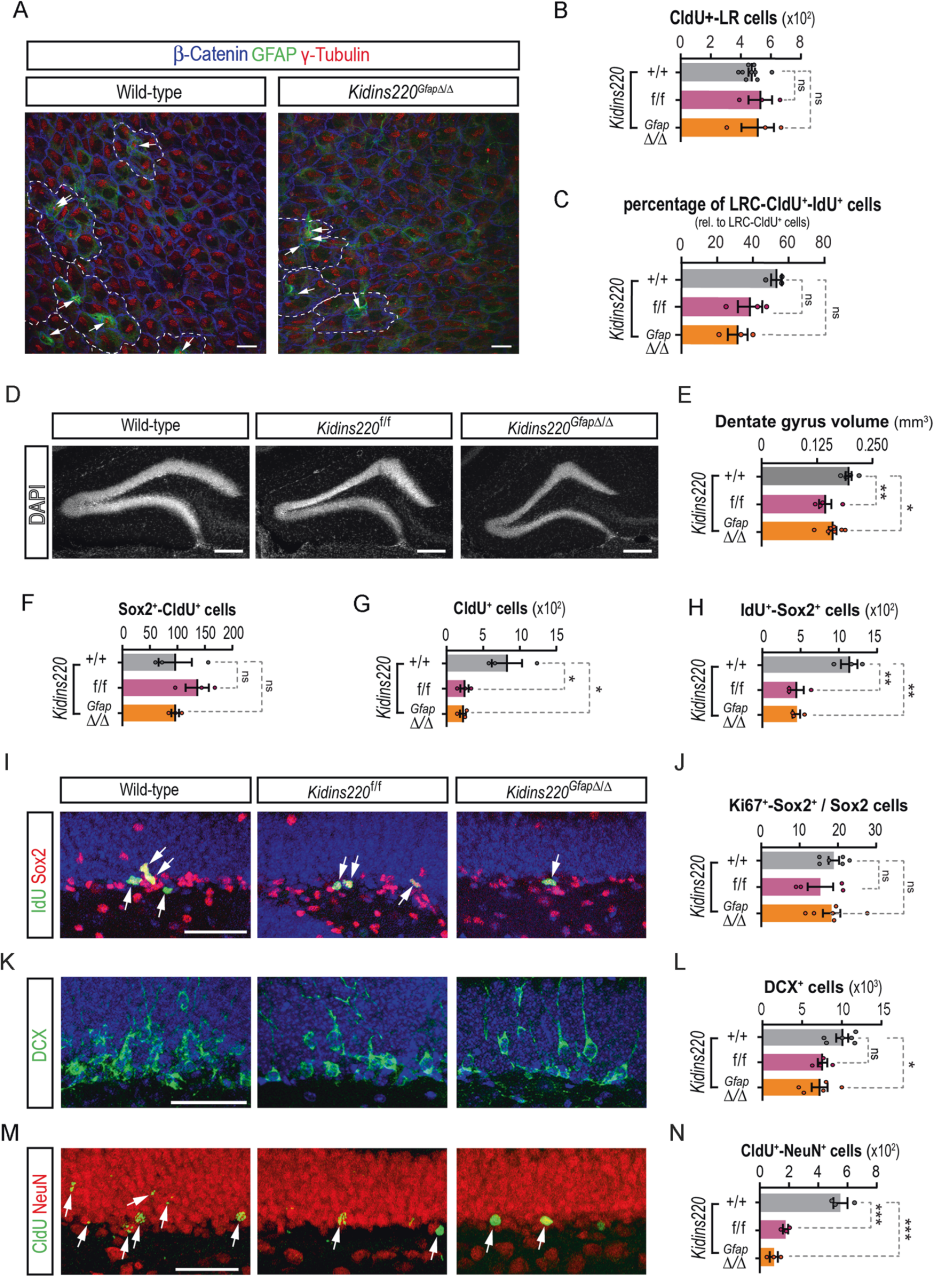
Neurogenic defects in Kidins220 deficient mice concentrate in the hippocampus. **A** Representative confocal images of wild-type and *Kidins220*^*Gfap*Δ/Δ^ lateral ventricle wholemount preparations stained for ß-catenin (blue), γ-tubulin (red) and GFAP (green) to reveal pinwheels (white dashed lines) and SEZ stem cells (arrows). Scale bar: 10 μm. **B**, Quantification of the numbers of CldU^+^-label retaining (LR) cells at the lateral ventricles of wild-type (*N* = 9), *Kidins220*^*f/f*^ (*N* = 3) and *Kidins220*^*Gfap*Δ/Δ^ (*N* = 3) mice. **C** Quantification of the percentage of IdU^+^ cells within the CldU^+^-LR cells at the lateral ventricles of wild-type (*N* = 3), *Kidins220*^*f/f*^ (*N* = 3) and *Kidins220*^*Gfap*Δ/Δ^ (*N* = 3) mice. **D** Confocal micrographs of the DG of wild-type, *Kidins220*^*f/f*^ and *Kidins220*^*Gfap*Δ/Δ^ mice. Nuclei are stained with DAPI (gray). Scale bar: 200 μm. **E**, Quantification of the DG volume (mm^3^) in wild-type (*N* = 5), *Kidins220*^*f/f*^ (*N* = 4) and *Kidins220*^*Gfap*Δ/Δ^ (*N* = 7) mice. **F**, Numbers of Sox2^+^- CldU^+^ cells, **G** CldU^+^ cells and **H**, IdU^+^-Sox2^+^ cells in the SGZ of wild-type, *Kidins220*^*f/f*^ and *Kidins220*^*Gfap*Δ/Δ^ mice (*N* = 3, for each condition). **I**, Representative confocal images of the co-staining for Sox2 (red) and IdU (green, arrows). Nuclei are stained with DAPI (blue). Scale bar: 50 μm. **J**, Percentage of Sox2^+^ cells positive for Ki67 in the SGZ of wild-type (*N* = 6), *Kidins220*^*f/f*^ (*N* = 4) and *Kidins220*^*Gfap*Δ/Δ^ (*N* = 6) mice. **K** Confocal images of the staining for the neuroblast marker doublecortin (DCX, green) and DAPI (blue). Scale bar: 50 μm. **L**, Quantification of the total numbers of DCX^+^ cells in wild-type (*N* = 6), *Kidins220*^*f/f*^ (*N* = 4) and *Kidins220*^*Gfap*Δ/Δ^ (*N* = 6) mice. **M** Representative confocal images of the staining for the neuronal marker NeuN (red) and CldU (green) 3 weeks after injection to label new-born neurons. Scale bar: 50 μm. **N** Quantification of CldU^+^-NeuN^+^ double positive cells in wild-type, *Kidins220*^*f/f*^ and *Kidins220*^*Gfap*Δ/Δ^ mice (*N* = 3, each). Data represent mean ± s.e.m.; each data point represents an individual mouse. ns, not significant, *P < 0.05, **P < 0.01, ***P < 0.001, by one-way ANOVA and Dunnett’s *post-hoc* test.

Kidins220 has been recently involved in the etiopathology of hydrocephalus, as *Kidins220*^*f/f*^ mice present ventriculomegaly at different degrees [23]. Similarly to floxed mice, *Kidins220*^*Gfap*Δ/Δ^ mice presented enlarged ventricles, as measured by magnetic resonance imaging (MRI), when compared to wild-type littermates (Fig. S3). To directly examine NSCs at the ventricular surface of Kidins220 mutants, we used whole-mount *en face* staining of NSCs. As previously described for *Kidins220*^*f/f*^ [23], *Kidins220*^*Gfap*Δ/Δ^ ventricular wall linings were apparently normal, showing multiciliated ependymal cells (Fig. 4A). In addition, we were able to detect NSCs exposed at the ventricle in *Kidins220*^*f/f*^ and *Kidins220*^*Gfap*Δ/Δ^ mice, with no overall changes in the pinwheel organization (Fig. 4A). To investigate whether low Kidins220 levels affect adult NSCs and neurogenesis, we injected 2-month-old wild-type and *Kidins220*^*f/f*^ mice with the thymidine analogue 5-chloro-2'-deoxyuridine (CldU) and kept them alive for 4 weeks, to label slow dividing NSCs at the SEZ, and new neurons at the OB, by CldU-retention [31] (see scheme and representative images of the stainings in Fig. S4). Constitutively reduced Kidins220 levels during development did not provoke adult NSC decreased seeding, since total number of NSCs, scored as CldU^+^-label retaining cells in the SEZ were unaltered in *Kidins220*^*f/f*^ *vs*. wild-type mice (Fig. 4B). As for *Kidins220*^*f/f*^, we did not find differences in the total number of NSCs in the SEZ of *Kidins220*^*GfapΔ/Δ*^ mice (Fig. 4B). We next performed cell counts of activated NSC (aNSC)/NSC-derived C cells by double labelling of CldU^+^-LRC with the analogue 5-iodo-2'-deoxyuridine (IdU) after 1 hour pulse [31] (Figs. 4C, S4). We did not find significant differences between genotypes, although a tendency towards a decrease could be observed in *Kidins220*^*GfapΔ/Δ*^ mice (Fig. 4C). However, at the age analysed, this tendency did not translate in vivo into reduced numbers of NSCs/progenitors labelled with Sox2^+^, Ki67^+^ cycling progenitors, in the neuronal-lineage marker doublecortin (DCX)^+^-neuroblast population, nor in the newly generated OB neurons (scored as CldU^+^ cells at the OB glomeruli) (Fig. S5A-H). Likewise, newly incorporated oligodendrocytes to the corpus callosum (CldU^+^ cells), and newly formed terminally differentiated astrocytes at the SEZ (CldU^+^-S100β^+^-double positive cells) [33] were unaltered (Fig. S5I-L), indicating that Kidins220 deficiency does not alter NSC-astrocytic terminal differentiation nor oligodendrogenesis, at the age studied. Histological examination of *Kidins220*^*f/f*^ and *Kidins220*^*GfapΔ/Δ*^ DG showed that cytoarchitectural organization was apparently preserved, although the volume was significantly smaller than that of wild-type mice (Fig. 4D, E). Similarly to the SEZ, Kidins220 deficiency did not alter the total number of NSC, scored as Sox2^+^-CldU^+^ cells, (Fig. 4F). However, we discovered a sharp decrease in the numbers of total CldU^+^ cells in the DG (Fig. 4G), suggesting a deficit in newborn neurons. By combining Sox2 and IdU stainings (injected 1 hour before sacrifice to label proliferative aNSC/progenitors [31]; Fig. S4A), we found a decrease in type 2 cells in Kidins220-deficient mice (Fig. 4H, I). This reduction was not due to impaired proliferation of aNSC/progenitors, since the rate of cycling (Ki67^+^)-Sox2^+^ cells was unaffected in *Kidins220*^*f/f*^ and *Kidins220*^*GfapΔ/Δ*^ compared to wild-type mice (Fig. 4J). Concomitantly, we found a reduction in the number of neuroblasts expressing DCX in *Kidins220*^*Gfap*Δ/Δ^ mice (Fig. 4K, L), albeit this decrease did not reach significance in *Kidins220*^*f/f*^ animals (Fig. 4K, L). We then determined the emergence of newborn neurons in mutant SGZs, scoring the percentage of CldU^+^ cells that had acquired the neuronal marker NeuN. We observed a sharp decrease in newly generated neurons in *Kidins220*^*f/f*^ and *Kidins220*^*GfapΔ/Δ*^ mice, indicating a neurogenic deficit in adulthood (Fig. 4M, N).

### Functional consequences of Kidins220 deficiency in hippocampal neurogenesis

**Fig. 5:**
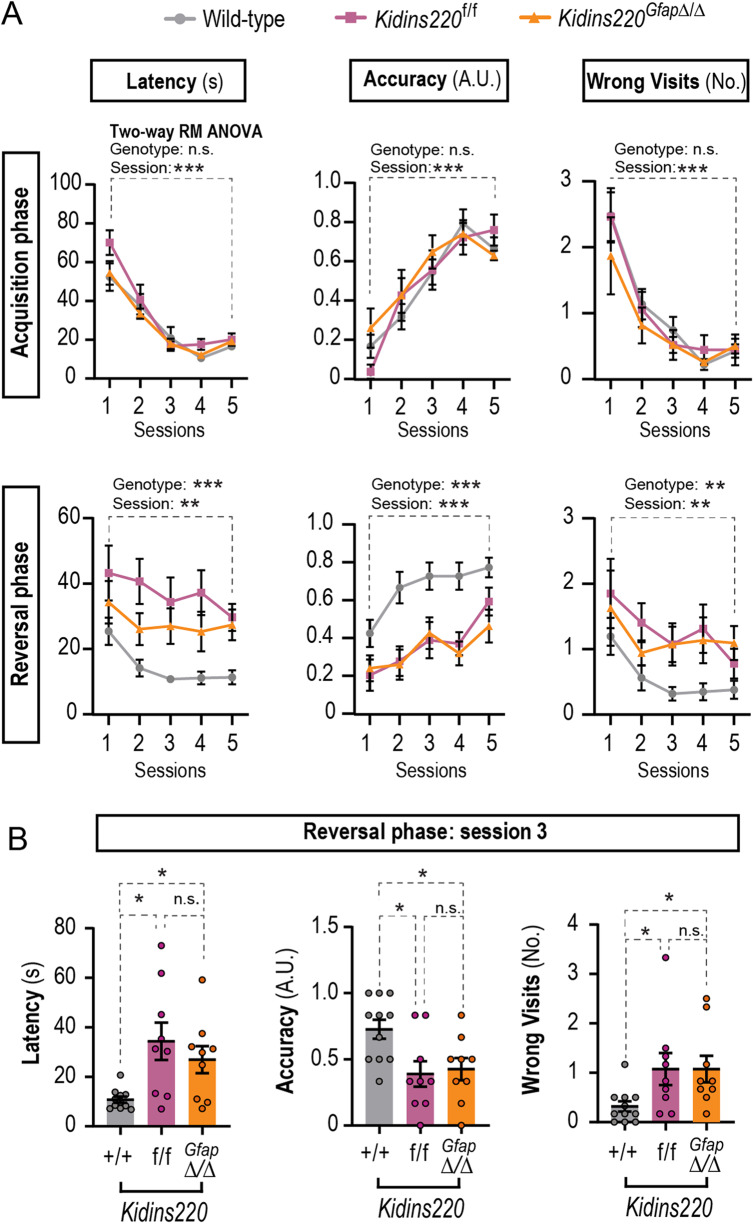
Kidins220-defficient mice exhibit deficits in spatial memory. **A** T maze test results during acquisition phase (upper panels) and reversal phase (lower panels). Wild-type (*N* = 11), *Kidins220*^*f*/f^ (*N* = 9) and *Kidins220*^*GfapΔ/Δ*^ (*N* = 9) mice were challenged for 5 sessions, with 6 trials in each session, and three parameters were scored: latency in rewarded arm (the time (s) to reach the reward and start eating), accuracy (arbitrary units: 1 for entering the rewarded arm and eating from the pellet within 30 seconds; 0 for any other outcome), and the number of visits to the non-rewarded arm (wrong visits). The effects of the genotype and sessions and their interaction were analyzed by two-way repeated measures (RM) ANOVA. Interaction was non-significant (*P* > 0.05). **B** One-way ANOVA analyses of data from session 3 in the reversal phase, for latency, accuracy and number of wrong visits for wild-type, *Kidins220*^*f*/f^ and *Kidins220*^*GfapΔ/Δ*^ mice. Data represent mean ± s.e.m.; each data point represents an individual mouse. Ns, not significant, *P < 0.05, **P < 0.01, ***P < 0.001, by two-way RM (**A**) or one-way ANOVA followed by Dunnett’s T3 (latency) or Tukey’s *post-hoc* tests (accuracy and wrong visits).

To analyze the functional consequences of the reduced adult hippocampal neurogenesis, we performed behavioral tests. To assess hippocampal-based spatial working memory, mice from the 3 genotypes were subjected to the T maze test (Fig. 5A, B). For the parameters scored during the first week (latency, accuracy, and visits to the unrewarded arm), results showed there was not a statistically significant interaction between the effects of genotype and sessions (Fig. 5A and see Table S3 for detailed ANOVA results). As expected, the variable ‘genotype’ did not have a statistically significant effect on spatial working memory whilst, the variable ‘session’ did, for all three parameters scored. This indicates that during the acquisition phase, all mice independently of their genotypes, showed a similar reduction in the number of wrong visits and latency, and a similar increase in accuracy, indicating a successful acquisition of T-maze discrimination learning throughout the sessions (Fig. 5A). In contrast, ‘genotype’ and ‘session’ had a statistically significant impact on reversal learning for the three parameters scored (Fig. 5A). This indicated that during the reversal phase, only wild-type mice showed a successful reversal learning, obtaining a good score of accuracy and a reduction of wrong visits and latency compared to *Kidins220*^*f/f*^ and *Kidins220*^*GfapΔ/Δ*^ mice. A closer examination of the data suggested that wild-type mice successfully achieved learning on the third session. In contrast, both *Kidins220*^*f/f*^ and *Kidins220*^*GfapΔ/Δ*^ mice displayed an increased latency in completing the trial and in the number of visits to the wrong arm, while showed a lack of accuracy in comparison with wild-type mice in session 3 (Fig. 5B). Taken together, these results suggest that physiological Kidins220 levels promote cognitive flexibility.

### Kidins220 deficiency decreases neural stem cell growth and survival

**Fig. 6:**
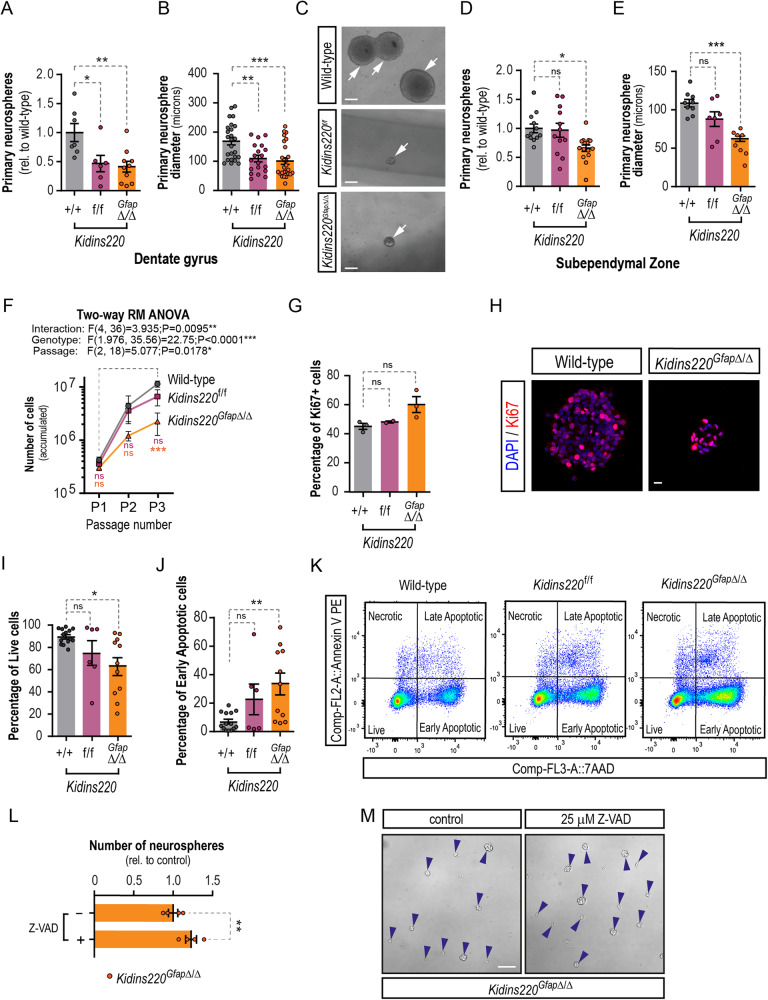
Viability defect in Kidins220-deficient neural stem cells. **A** Number of primary neurospheres obtained from the hippocampus of wild-type (*N* = 7), *Kidins220*^*f/f*^ (*N* = 6), and *Kidins220*^*Gfap*Δ/Δ^ (*N* = 10) mice. **B** Diameter of wild-type (*N* = 25), *Kidins220*^*f/f*^ (*N* = 19), and *Kidins220*^*Gfap*Δ/Δ^ (*N* = 26) primary hippocampal neurospheres from *N* = 3 mice from each genotype. **C** Representative phase contrast images of primary hippocampal neurospheres from wild-type, *Kidins220*^*f/f*^, and *Kidins220*^*Gfap*Δ/Δ^ mice. Scale bar, 50 μm. **D** Number of primary neurospheres obtained from the walls of the lateral ventricles of wild-type (*N* = 12), *Kidins220*^*f/f*^ (*N* = 12), and *Kidins220*^*Gfap*Δ/Δ^ (*N* = 15) mice. **E**, mean neurosphere diameter of SEZ neurospheres of wild-type (*N* = 9), *Kidins220*^*f/f*^ (*N* = 7) and *Kidins220*^*Gfap*Δ/Δ^ (N = 8) mice. **F**, Cumulative number of cells obtained over 3 consecutive passages of SEZ neurospheres (*N* = 7 independent cultures *per* genotype). **G** Percentage of Ki67^+^ cells in SEZ neurospheres of the three genotypes (wild-type, *N* = 3; *Kidins220*^*f/f*^, *N* = 2; *Kidins220*^*Gfap*Δ/Δ^, *N* = 3). **H** Representative confocal micrographs of wild-type and *Kidins220*^*Gfap*Δ/Δ^ SEZ neurospheres stained for the cell cycle marker Ki67 (red) and DAPI (blue) to counterstain the nuclei. Scale bar: 10 μm. **I** Quantitative analysis of the percentage of live cells (Annexin V^-^/7AAD^-^) and **J**, of early-stage apoptotic cells (Annexin V^+^/7AAD^-^) in wild-type (*N* = 14), *Kidins220*^*f/f*^ (*N* = 6) and *Kidins220*^*Gfap*Δ/Δ^ (*N* = 11) NSCs from the SEZ. **K** Representative FACS dot-plots of SEZ NSC double-stained for Annexin V/7-AAD. **L**, Quantification of the number of SEZ neurospheres from *Kidins220*^*Gfap*Δ/Δ^ formed after a treatment with 25 μM Z-VAD to inhibit caspases or vehicle for 4 days (*N* = 4 independent experiments). **M** Representative phase contrast micrographs of *Kidins220*^*Gfap*Δ/Δ^ SEZ neurospheres in both conditions, scale bar: 50 μm. Data represent mean ± s.e.m. Each data point represents values from a neurosphere culture established from an individual mouse (except in **B**), or an individual mouse (**O**). Ns, not significant, *P < 0.05, **P < 0.01, ***P < 0.001, by two-tailed paired t test (L), one-way ANOVA (**A**, **B**, **D**, **E**, **G**, **I**, **J**) and two-way RM ANOVA (**F**) followed by Dunnett’s *post-hoc* tests. 7AAD, 7-aminoactinomycin D; FACS, fluorescence-activated cell sorting.

Fresh tissue from both neurogenic regions can be dissected, homogenized and cultured in vitro as free-floating aggregates of NSCs and progenitors known as neurospheres [34, 35], or alternatively grown in adherent conditions seeded on a laminin-rich substrate [36]. In both cases, in the presence of epidermal growth factor (EGF) and fibroblast growth factor-2 (FGF-2) as main mitogens, NSCs derived from the SEZ and the DG thrive and proliferate over several passages maintaining their multipotency. Importantly, NSCs cultured in vitro expressed detectable levels of Kidins220 (Fig. S6A, C). Furthermore, hippocampal NSCs obtained from *Kidins220*^*f/f*^ and *Kidins220*^*GfapΔ/Δ*^ grown in vitro yielded significantly less primary neurospheres (Fig. 6A), and of substantially smaller diameter than their wild-type counterparts (Fig. 6B, C), showing that there is a striking defect in the ability to form hippocampal-derived neurospheres of adequate size from Kidins220-deficient mice. Accordingly, attempts of expanding these cultures beyond the first passage failed (Fig. S6D), corroborating in vivo data showing the extreme sensitivity of the SGZ to Kidins220 levels, and unveiling a cell-autonomous effect in NSCs derived from this niche. SEZ-derived neurospheres from *Kidins220*^*f/f*^ mice expressed half the levels than wild-type, while Kidins220 levels were undetectable in *Kidins220*^*GfapΔ/Δ*^ cells (Fig. S6B). Despite the detection of normal numbers of NSCs in the intact SEZ of *Kidins220*^*GfapΔ/Δ*^ mice, we obtained reduced numbers of neurosphere-initiating cells (Fig. 6D) indicating a cell-autonomous defect as well, not obvious in vivo at the age studied. In contrast, *Kidins220*^*f*/*f*^ SEZs did not display obvious differences in primary neurosphere output (Fig. 6D), suggesting that a certain level of Kidins220 is required for NSCs to thrive in vitro (Fig. S6B). *Kidins220*^*GfapΔ/Δ*^ SEZ cells could be passaged but rendered neurospheres with markly diminished diameter (Fig. 6E) and yielded reduced accumulated cell numbers *vs*. wild-type and *Kidins220*^*f/f*^ cells (Fig. 6F). This phenotype was not accompanied by substantial defects in proliferation rate (Fig. 6G, H), altogether suggesting a viability defect. Staining for 7-aminoactinomycin D (7AAD) and Annexin V, to assess the percentage of live and apoptotic cells in NSC cultures by flow cytometry, showed that *Kidins220*^*Gfap*Δ/Δ^ cells had an overall decreased survival and a higher rate of apoptosis than wild-type, affecting as much as the 65% of the culture, hence suggesting a cause for NSC depletion. (Fig. 6I–K). Accordingly, treatment with the pan-caspase inhibitor of apoptosis Z-VAD-FMK rescued the defect in neurosphere formation in *Kidins220*^*GfapΔ/Δ*^ cells (Fig. 6L, M).

### Kidins220 is necessary to suppress GSK3-mediated neural stem cell and progenitors death, downstream of growth factor signaling

**Fig. 7:**
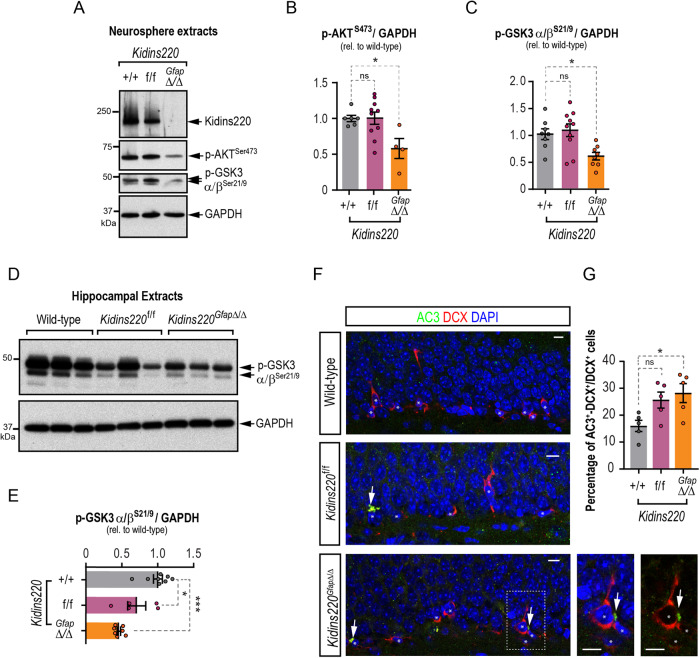
Kidins220 deficiency impairs AKT/GSK3 survival pathway in neural stem cells and in the hippocampus, rendering increased death of neuronal progenitors in the SGZ. **A** Representative immunoblots for Kidins220, p-AKT^Ser473^, p-GSK3α/β^Ser21/9^ and GAPDH in lysates from *Kidins220*^*Gfap*Δ/Δ^, *Kidins220*^*f/f*^ and wild-type neurospheres. Levels of p-AKT^S473^ (wild-type, *N* = 6; *Kidins220*^*f/f*^, *N* = 10; *Kidins220*^*Gfap*Δ/Δ^, *N* = 3) (**B**) and p-GSK3α/β^S21/9^ (wild-type, *N* = 8; *Kidins220*^*f/f*^, N = 10; *Kidins220*^*Gfap*Δ/Δ^, *N* = 8) (**C**) represented in arbitrary units after normalization with GAPDH, and relative to wild-type in extracts from the indicated genotypes. **D** Representative immunoblots for p-GSK3α/β^Ser21/9^ and GAPDH in hippocampal lysates from wild-type, *Kidins220*^*f/f*^ and *Kidins220*^*Gfap*Δ/Δ^ mice. **E** Levels of p-GSK3α/β^S21/9^ represented in arbitrary units after normalization with GAPDH and relative to wild-type in extracts from the indicated genotypes (wild-type, *N* = 8; *Kidins220*^*f/f*^, N = 5; *Kidins220*^*Gfap*Δ/Δ^, *N* = 7). **F**, Confocal images of the staining of DCX (red), active caspase 3 (AC3, green) and DAPI (blue) in the SGZ of wild-type, *Kidins220*^*f/f*^ and *Kidins220*^*Gfap*Δ/Δ^ mice (*N* = 5 mice of each genotype). Scale bar: 5 μm. **G** Quantification of the percentage of DCX^+^ cells positive for AC3 in the SGZ of wild-type, *Kidins220*^*f/f*^ and *Kidins220*^*Gfap*Δ/Δ^ mice (*N* = 5 mice of each genotype). Data represent mean ± s.e.m. Each data point represents values from a neurosphere culture established from an individual mouse (**B**, **C**), brain extracts from an individual mouse (**E**) or brain immunolabeling from an individual mouse (**G**). Ns not significant, *P < 0.05, **P < 0.01, ***P < 0.0001, by one-way ANOVA followed by Dunnett’s (**B**, **C**, **E**, **G**) *post-hoc* tests.

The interaction of various growth factors with their respective receptor tyrosine kinases activates the PI3K/AKT pro-survival pathway. Activated AKT can then phosphorylate the two GSK3 isoforms on their N-terminus (at Ser21 for GSK3α and Ser9 for GSK3β), inactivating them [37, 38]. Suppression of GSK3 activity by PI3K/AKT signaling is indeed the major pathway through which its pro-apoptotic roles are prevented [39]. Given the survival defect of *Kidins220*^*Gfap*Δ/Δ^ neurospheres, we investigated whether the PI3K/AKT signaling pathway was specifically affected by performing immunoblots for the activating phosphorylation of AKT at serine 473 (AKT^pSer473^) [40] and subsequent phosphorylation of GSK3α/β^pSer21/9^. We found a significant reduction in active AKT, as well as in the inhibitory phosphorylation of GSK3 isoforms in *Kidins220*^*Gfap*Δ/Δ^ neurospheres (Fig. 7A–C). Likewise, we found a decrease in active AKT in lysates from hippocampal neurospheres (Fig. S6E). Given the severe deficit in hippocampal progenitors, neuroblasts and newborn neurons observed in vivo, we hypothesized an over-activation of GSK3 in the hippocampus of Kidins220 genetic models and observed a marked deficiency of inactivating phosphorylations in both *Kidins220*^*f*/f^ and *Kidins220*^*Gfap*Δ/Δ^ hippocampal lysates compared to wild-types (Fig. 7D, E). These data indicate that Kidins220 fine-tunes the PI3K/AKT pro-survival pathway in response to external signals in NSC. We next analyzed whether decreases in this pro-survival pathway were accompanied by cell death in the hippocampal niche. Since no differences were observed in vivo in the number of DG NSCs in *Kidins220*^*GfapΔ/Δ*^ and *Kidins220*^*f/f*^ mice, but it was so in more committed progenitors and differentiated progeny, we focused our analysis of cell death in vivo on DCX^+^ neural progenitors, by combining DCX and active caspase-3 (AC3) immunostainings. We found an increased proportion of AC3^+^ cells within the DCX^+^ population in *Kidins220*^*GfapΔ/Δ*^ and a similar tendency in *Kidins220*^*f/f*^ mice, when compared with wild-type animals (Fig. 7F, G).

### EGF-receptor activation is impaired in Kidins220-null neural stem cells and can be rescued by inhibiting GSK3

**Fig. 8:**
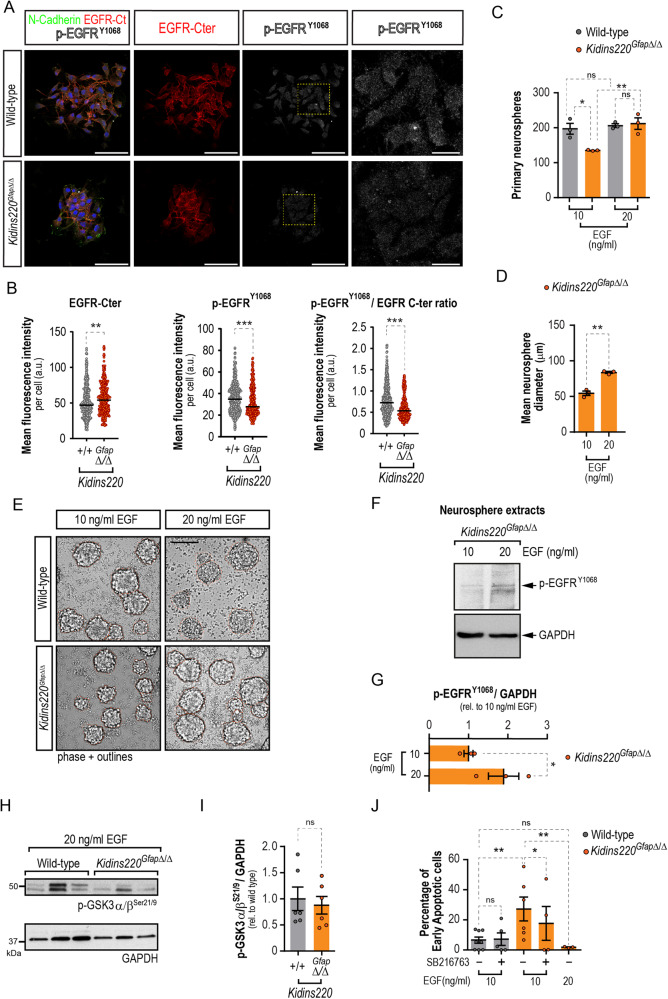
Recuperation of GSK3 inhibition downstream EGF-receptor restores survival defect in Kidins220-null neural stem cells. **A** Representative confocal micrographs of wild-type and *Kidins220*^*Gfap*Δ/Δ^ neurospheres stained for N-Cadherin (green), total EGFR C-terminal (Cter) domain (red), activated phospho-EGFP^Y1068^ (p-EGFR^Y1068^) (gray) and DAPI (blue) to counterstain the nuclei. Scale bar: 50 μm, and 12 μm (insets). **B** Quantification of the mean fluorescence intensity *per* cell of EGFR-Cter domain, p-EGFP^Y1068^, and the phosphorylated/total EGFR ratio. **C** Numbers of primary neurospheres obtained from wild-type and *Kidins220*^*Gfap*Δ/Δ^ mice under low (10 ng/ml) or high (20 ng/ml) EGF mitogenic stimulation (*N* = 3, for each condition). **D**, Quantification of the diameters of *Kidins220*^*Gfap*Δ/Δ^ neurospheres grown under low and high EGF (*N* = 3, for each condition). **E**, Representative phase contrast micrographs of wild-type and *Kidins220*^*Gfap*Δ/Δ^ primary neurospheres grown in culture medium with EGF 10 or 20 ng/ml. Neurosphere borders are outlined in orange for clarity. Scale bar 100 μm. **F** Representative immunoblots for p-EGFR^Y1068^ and GAPDH in lysates from *Kidins220*^*Gfap*Δ/Δ^ neurospheres grown in 10 and 20 ng/ml EGF. **G** Levels of p-EGFR^Y1068^ represented in arbitrary units after normalization with GAPDH and relative to values from 10 ng/ml EGF condition (*N* = 3 for each condition). **H** Representative immunoblots for p-GSK3α/β^Ser21/9^ and GAPDH in lysates from *Kidins220*^*Gfap*Δ/Δ^ and wild-type primary neurospheres grown in 20 ng/ml EGF. **I** Levels of p-GSK3α/β^S21/9^ represented in arbitrary units after normalization with GAPDH and relative to wild-type in extracts from the indicated genotypes (*N* = 6, for each condition). **J**, Quantification of the percentage of apoptotic cells in 7-AAD and Annexin V staining detected by flow-cytometry. Wild-type and *Kidins220*^*Gfap*Δ/Δ^ neurospheres were grown in 10 or 20 ng/ml EGF and treated with 100 nM SB216763 GSK3 inhibitor or vehicle (*N* = 8, 5: wild-type, 10 ng/ml EGF, vehicle or SB216763, respectively; *N* = 6, 4: *Kidins220*^*Gfap*Δ/Δ^, 10 ng/ml EGF, vehicle or SB216763, respectively; *N* = 3: *Kidins220*^*Gfap*Δ/Δ^, 20 ng/ml EGF). Data represent mean ± s.e.m. Each data point represents values from a neurosphere culture established from an individual mouse (**C**, **D**, **G**, **I**, **J**) or an independent cell (*N* = 437 for wild-type and 300 for *Kidins220*^*Gfap*Δ/Δ^ independent cells from neurosphere cultures established from 3 individual mice for each genotype, **B**). Ns, not significant, *P < 0.05, **P < 0.01, ***P < 0.0001, unpaired two-tailed Mann Whitney test (**B**), one-way ANOVA with Bonferroni *post-hoc* test (**C**), two-tailed paired (**D**, **G**, **J**) and unpaired t-tests (**I**, **J**).

The main growth factor/mitogen in neurosphere culture is EGF, so we hypothesized that *Kidins220*^*Gfap*Δ/Δ^ NSCs may display decreased levels of EGFR or downstream signaling. We performed immunofluorescence and confocal microscopy image analysis in wild-type and *Kidins220*^*GfapΔ/Δ*^-derived neurospheres, using anti-N-cadherin (as a cell membrane marker to localise the staining and be able to demarcate individual cell within the neurospheres), anti-EGFR C-terminal domain (to label the total amount of receptor) and antiphospho-EGFR^Y1068^ to detect its activated form (Fig. 8A). As shown, Kidins220 deficiency did not decrease the total levels of the receptor, and even caused a slight increase, but in contrast it provoked a clear decrease in EGFR activity, even more strikingly observed as the ratio of the total and phosphorylated signals (Fig. 8B). Our data indicate that EGFR-signalling is compromised in neurospheres lacking Kidins220.

Next, we hypothesized that increasing the concentration of EGF, could rescue the cell survival defect of *Kidins220*^*Gfap*Δ/Δ^ NSCs. *Kidins220*^*Gfap*Δ/Δ^ SEZ tissue seeded in medium with a higher EGF concentration (20 ng/ml) yielded ~30% more primary neurospheres than when seeded in low EGF (10 ng/ml), rising to wild-type numbers, which were unaffected by increasing EGF (Fig. 8C). The higher dose of EGF augmented the diameter of *Kidins220*^*Gfap*Δ/Δ^ neurospheres by ~40% (Fig. 8D, E). Finally, to test whether increasing EGF concentration in culture could increase EGFR-mediated signaling, we performed Western blots for phosho-EGFR^Y1068^ in lysates from *Kidins220*^*Gfap*Δ/Δ^ treated with low and high EGF doses. We found that al 10 ng/ml, EGF signaling was not yet saturating and that increased phosphorylation of the receptor could be achieved at 20 ng/ml (Fig. 8F, G). To test if enhancing signaling through EGF restored GSK3 inhibitory phosphorylation to wild-type levels, we performed immunoblots in extracts from neurospheres grown in high EGF and found no significant differences between genotypes (Fig. 8H, I). Next, we analyzed whether increasing EGF concentration could rescue cell viability. Culturing *Kidins220*^*Gfap*Δ/Δ^ cells in 20 ng/ml EGF rescued the enhanced apoptosis found when cultured in 10 ng/ml EGF, decreasing cell death (Fig. 8J). Furthermore, treatment of wild-type and *Kidins220*^*Gfap*Δ/Δ^ cells with the GSK3 inhibitor SB216763 could still circumvent cell apoptosis in *Kidins220*^*Gfap*Δ/Δ^ cells, although to a lesser extent than a high dose of EGF (Fig. 8J).

## Discussion

In this work we have identified Kidins220 as a critical mediator of hippocampal adult neurogenesis, and a novel intrinsic regulator of NSCs and progenitors, that plays a role tuning PI3K/AKT pro-survival signaling in response to mitogenic stimulation. Mechanisms of survival in adult NSC populations are barely known, even less those that may be related to mitogens that stimulate their division, as survival is a prerequisite for proliferation. Our data indicate that the level of Kidins220 sets the threshold of mitogen-induced activation that sustains adult NSC survival.

*Kidins220* is an essential gene and its global deletion causes extensive cell death in the developing nervous system, accompanied by a growth defect, with embryos displaying smaller bodies and brain size [14, 15]. We show that Kidins220 is abundantly expressed in NSCs from the SEZ and SGZ and that hippocampal neurogenesis is drastically dependent on Kidins220 levels. Neurogenesis in the DG starts during embryonic development and continues through adulthood with the addition of newborn granule neurons [41]. Since Kidins220 deletion using a *Nes*-Cre driver does not cause increased cell death in the embryonic hippocampus at E18.5, when cells accumulate in the DG [14, 15], the effect of Kidins220 deficiency on hippocampal development appears to be mainly postnatal. Postnatal effects of Kidins220 deficiency can account for the reduced volume of DG found in both genetic models, since the DG is formed from E20 to P5 [41]. The effects of Kidins220 global deficiency on hippocampal formation during postnatal development deserve future investigation.

A reduction in Kidins220 markedly decreases type 2 cycling progenitors and the concomitant emergence of DCX^+^ neuroblasts and adult-born neurons, indicating that survival might be compromised in NSC-derived progeny when Kidins220 levels drop in the DG. Indeed, we found an increase in neuroblasts with immunoreactivity of activated caspase 3 indicative of their induced apoptotic death. Accordingly, Kidins220 deletion in immature hippocampal neurons leads to multiple axons and dendritic aberrations [42], whereas its silencing in mature cortical cultured neurons increases their death and vulnerability to excitotoxicity [16, 18]. Notably, the defects we identify herein correlate with impaired hippocampal-dependent spatial working memory, a trait strongly associated with the depletion of adult hippocampal neurogenesis [43]. The sharp decline in hippocampal neurogenesis with advancing age is associated to age-related cognitive deficits due to its function in learning and memory. As in Kidins220-deficient mice, neurogenesis at the DG of aged mice is substantially reduced relative to the SEZ [44], consistent with a potential premature aging of neurogenic niches in these models. Importantly, administration of growth factors restored aging-associated neurogenic defects in both niches [44]. Similarly, our data show that *Kidins220*^*GfapΔ/Δ*^ SEZ-derived neurospheres are rescued by extrinsic cues, as their number, size, and pro-survival pathways are restored to wild-type values by increasing the concentration of EGF.

The extreme sensitivity of DG neurogenesis to reduced Kidins220 levels is in contrast with our findings from the SEZ, which seems refractory to Kidins220 deficiency. This fact may indicate that, in addition to the cell-autonomous effect described, the hippocampal niche may be altered in Kidins220-deficient mice and does not provide the adequate cues, a possibility that we have not explored herein. In our analysis of the SEZ we found a tendency to decrease NSC activation in the SEZ in vivo. SEZ aNSC are highly dependent on EGFR [8], and decreased activation of NSC is a hallmark of aging [45] so we cannot rule out that in older animals Kidins220-deficiency would lead to a premature aging phenotype.

Nevertheless, the fact that NSCs from both niches show a cell-autonomous defect in survival ex vivo, suggests that the niche microenvironment is promoting NSCs survival in the absence of Kidins220. The role of the niche in the maintenance of NSCs and derived progeny is well known [4, 46]. One possibility is that the levels of TGFα, the endogenous EGFR ligand, are sufficient to sustain Kidins220-deficient NSCs survival in vivo. However, we found a deficit in neuroblast survival in the SGZ *vs*. the SEZ, where no phenotype was observed at the age studied. EGFR expression is very prominent in the SEZ compared to the lower levels of this receptor in the SGZ, where NSCs respond less to EGF [47]. In addition, both adult neurogenic niches present differential susceptibility to apoptotic stimuli. For instance, high FOXG1 levels hinders the survival of SGZ-derived glutamatergic dentate granule neurons, but spares generation of GABAergic neurons in the SEZ/OB [48]. Thus, reducing Kidins220 levels could affect more severely the hippocampal NSC-derived populations than those from the SEZ, due to their lower content, and/or that neuroblasts committed to glutamatergic neurons are more sensitive to Kidins220 deficiency than GABAergic ones.

Our data support the notion that Kidins220 is crucial for cell survival of neurons and precursors, without majorly affecting their proliferation. Neurotrophin signaling may be key in Kidins220-mediated control of DG ontogenesis and adult neurogenesis, since Kidins220 has been shown to interact with all Trk-receptors and with p75NTR [49, 50], and reduced neuronal numbers may be due to decreased trophic support in Kidins220-deficient mice by diminished response to neurotrophins.

Kidins220-deficient mice present hydrocephalus [23], and because previous reports have linked reduced SEZ NSCs numbers to this condition in vivo [51, 52], we have only investigated mice with moderate enlargement of the ventricles (see Fig. S3) in this study. Our data suggest that the milder presentations of ventriculomegaly do not affect SEZ NSCs and progeny, at least in our Kidins220-deficient mice models and at the age studied. Our data call for further investigations on the SEZ in Kidins220-deficient mice afflicted with severe hydrocephalus, as future follow-up for this work.

Keeping Kidins220 levels over a certain threshold maintains PI3K/AKT-mediated GSK3 inhibition in NSCs and hippocampal extracts in both Kidins220-deficient mice strains, and increased dosage of EGF rescues cell survival in NSCs restoring GSK3 inhibition. EGFR does not co-immunoprecipitate with Kidins220 [50], so the possibility of Kidins220 regulating the clustering of this receptor in NSCs is unlikely. However, Kidins220 may modulate a downstream EGFR effector. Remarkably, Kidins220 provides a molecular docking site for the CrkL adaptor protein to sustain mitogen-activated protein kinases/extracellular signal-regulated kinases signaling in neurons in response to nerve growth factor [53]. A parallel mechanism might play a role in NSCs, as CrkL directly binds to and regulates PI3K/AKT [54–56]. Since silencing Kidins220 does not alter AKT activation in cultured neurons [50], Kidins220 may be crucial for PI3K/AKT activation downstream EGFR signaling in activated NSCs and neuronal progenitors, in contrast to terminally differentiated neurons. EGFR can induce PI3K/AKT/mTOR and MEK/ERK cascades in cultured neural stem and progenitor cells (NSPCs), but only the former implicating mTOR contributes to their survival [57]. In this context, and with the results we provide herein, it is likely that Kidins220 is involved in the potentiation of this PI3K/AKT/mTOR prosurvival pathway induced by EGFR stimulation in NSPCs.

A growing body of evidence points to a direct role of KIDINS220 in cancer [58]. In castration-resistant prostate cancer, KIDINS220 activates the PI3K/AKT pathway [59]. Importantly, in this type of cancer there is a high prevalence of activating PI3K/AKT/mTOR pathway alterations linked to tumor cell survival and tumorigenesis [60], again highlighting the possible role of Kidins220 in cell viability through this cascade. Additionally, in pediatric high-grade glioma copy number breakpoint within *KIDINS220* gene have been identified [61]. NSCs of the SEZ are considered glioma cells of origin [62, 63], and molecular alterations of the EGFR/PI3K/AKT/mTOR module are hallmarks of this cancer type, which has led to the design of clinical trials devised to target this pathway [64]. Whether KIDINS220 could be part of the stimulus-response threshold activating this pro-survival pathway downstream of EGFR in glioma stem cells and possible therapeutic implications, deserves further investigation.

Maintaining the proper levels of GSK3 activity is crucial for neural progenitor maintenance, proliferation, survival, and differentiation [65, 66]. While GSK3 inhibition promotes adult hippocampal neurogenesis [67, 68], increased GSK3 activity reduced DG volume and causes defects in neuroblast generation, features like those reported herein [69]. Consistently, failure to properly regulate GSK3 activity plays a role in Alzheimer’s disease and schizophrenia [70–72], diseases associated with Kidins220 alterations, amongst others [17, 21–28, 72]. Exciting new studies support the idea that neurogenic potential preservation may be crucial to restrain cognitive decline associated with neurodegenerative conditions and non-physiological aging in humans [73, 74]. The data presented herein point to the possibility that alterations in KIDINS220 could render the DG neurogenic niche more sensitive to cognitive decline, and memory and learning disabilities, traits associated with these diseases. In summary, our data pinpoints Kidins220 as a multifaceted molecule with relevant functions in NSCs and adult neurogenesis.

## Materials and Methods

### Mice

*Kidins220*^*f*/f^ mice have been described previously [14, 15, 23]. B6.Cg-Tg(Gfap-cre)73.12Mvs/J (herein referred to as Gfap-Cre) were purchased from The Jackson Laboratory, (Bar Harbor, Maine, USA; JAX stock #012886) [32]. All animals were produced and housed at the animal care facility at the Instituto de Investigaciones Biomédicas Alberto Sols (IIBM, CSIC-UAM, Madrid, Spain) and maintained under 12/12 h light-dark cycle and with access to food and water ad libitum in a temperature-controlled environment. Overall mouse health was assessed by daily inspection for signs of discomfort, weight loss, or changes in behaviour, mobility, and feeding or drinking habits. Housing of mice and all experiments were conformed to the appropriate national legislations (RD 53/2013) and the guidelines of the European Commission for the accommodation and care of laboratory animals (revised in Appendix A of the Council of Europe Convention ETS123), following protocols approved by the corresponding local ethics committees (IIBM and CSIC). Male and female 2-month-old *Kidins220*^*f*/f^, *Kidins220*^*Gfap*Δ/Δ^ or wild-type littermates were employed. Genotyping and recombination were monitored in genomic DNA samples by PCR using specific pairs of primers (Table S1). For long-term label retention experiments, mice were injected and brains collected and processed, or fresh tissue was dissected to obtain whole mounts of the SEZ as described elsewhere [30, 31].

### Antibodies

Detailed information about all antibodies and dilutions used for the different applications is given in Supplementary Information (Table S2).

#### Immunohistochemistry of mouse brain samples

Immunostainings were performed in free-floating sections, incubated for 48 h with the appropriate primary antibodies (Table S2). For the specific detection of the synthetic nucleosides, a 20 min 2 N HCl denaturalization followed by neutralization in borate buffer was performed before the addition of the antibodies. Immunofluorescent detections were performed with Alexa Fluor (Invitrogen) conjugated secondary antibodies. DAPI (1 μg/ml) was used for counterstaining. Images were acquired under the same settings for each experiment with Fluoview FV10i (Olympus, Shinjuku, Tokyo, Japan), LSM 710, (Zeiss, Germany), Leica TCS SP5 (Leica Microsistemas SLU, L’Hospitalet de Llobregat, Spain), LSM710 and LSM900 (Zeiss, Oberkochen, Germany) confocal laser scanning microscopes at the Optical and Confocal Microscopy Unit of the CBMSO (CSIC-UAM), IIBM confocal facility (SEMOC, CSIC-UAM), Servicio Interdepartamental de Investigación (SIdI) UAM or UV. Images were processed using ImageJ/Fiji open-source software package (v1.54d; National Institutes of Health, Bethesda, MD).

#### Cell Counting and dentate gyrus volume

Numbers of NSC at the ventricle surface, CldU^+^ cells in the SEZ, the OB and the corpus callosum were obtained as described previously [31, 75]. Double stainings for CldU and IdU were performed as described elsewhere [31]. For Mash1 stainings, tissue sections were subjected to an antigen retrieval procedure with citrate buffer. The total volume of the DG and the number of CldU^+^ cells, CldU^+^-Sox2^+^ NSC, precursor cells (IdU^+^-Sox2^+^), immature neurons (DCX^+^), and mature neurons (CldU^+^-NeuN^+^) were calculated using the physical dissector method adapted for confocal microscopy as described elsewhere [75]. Briefly, DG area was measured in each slice using ImageJ software in one confocal series composed of every fifth section of the whole DG. The DG volume was calculated as the area obtained × thickness of the slice (50 μm), and the total volume of the DG was calculated as the summatory of the volume ×5. Numbers of positive cells for each marker were scored in every plane of one confocal series composed of every sixth section of the whole DG. To obtain total cell numbers, data were multiplied ×6.

#### Behavioral tests

Habituation was performed by handling 5 min every other day on the previous week to the test. For the T maze test, mice were food restricted for 24 h before the beginning of the test, which consisted of an acquisition phase and a reversal phase. Only one of the two short arms was rewarded with food. For each trial, the mouse was placed in the ‘start’ section of the long arm and given a maximum of three minutes to complete the test. In the second phase of the experiment, reversal learning was tested and the contingency was reversed, i.e., the other short arm was rewarded. Acquisition and reversal phases lasted 5 days (6 daily trials, with a two-day break between both phases). We scored the number of visits to the non-rewarded arm (wrong visits), the time to reach the reward and start eating (latency), and accuracy (1 point was given for entering the rewarded arm and eating the pellet within 30 seconds; any other option was scored with 0 points).

### Magnetic resonance imaging

Magnetic resonance imaging (MRI) was performed as described [23]. Briefly, MRI studies were performed in a Bruker Biospect 7.0-T horizontal-bore system (Bruker Medical Gmbh, Ettlingen, Germany), equipped with a 1H selective birdcage resonator of 23 mm and a Bruker gradient insert with 90 mm of diameter (maximum intensity 36 G/cm). Data were acquired using a Hewlett-Packard console running Paravision software (Bruker Medical Gmbh) operating on a Linux platform.

#### Cell culture, immunofluorescence and cell viability analysis by flow cytometry

Neurosphere cultures were established and maintained as described with minor modifications [34, 35] or were cultured in adhesive conditions as described previously [36] using Geltrex (Invitrogen, Waltham, MA, USA,) as coating substrate, with murine natural EGF (10 or 20 ng/ml, Thermofisher Scientific, Waltham, MA, USA) and human recombinant FGF-2 (10 ng/ml; Merck Millipore, Burlington, MA, USA) as mitogens. Cells were treated with 25 μM Caspase Inhibitor Z-VAD-FMK (carbobenzoxy-valyl-alanyl-aspartyl-[O-methyl]-fluoromethylketone, Promega Biotech Ibérica, Madrid, Spain) for 4 DIV. Primary neurosphere numbers and diameters were assessed by manual counting and photographed in an inverted microscope (Axiovert200, Zeiss ORCA-Flash4.0 LT sCMOS, Hamamatsu Photonics, Hamamatsu, Japan), and diameters measured using ImageJ/FIJI (NIH). For immunofluorescence, neurospheres were allowed to attach to Geltrex for 10 min, fixed with PFA 4%, and antibody staining was performed as previously described [76]. For bioimage analysis, images were acquired with an LSM900 confocal laser scanning microscope coupled to an upright microscope Axio Imager 2 (Zeiss). Laser settings were first established on wild-type samples and kept throughout the whole experiment. Random neurospheres consisting of 15-30 cells of each condition were imaged. Bioimage analysis was performed using ImageJ/Fiji. Cytoplasmic binary masks for each cell were obtained from the N-Cadherin signal previously subjected to an image processing and each cell was identified as an independent region of interest (ROI). The cytoplasmic binary masks were redirected to the original grayscale images and the mean gray intensities of EGFR-C-terminal and p-EGFR^Y1068^ were measured with Fiji software. Intensities were represented as the mean gray value in arbitrary units per cell in each comparison. Culture of primary cortical astrocytes was performed as described [23]. For flow cytometry, and rescue experiments, cells were cultured with 10 or 20 ng/ml EGF for three passages before cells were seeded on Geltrex-coated plates with the appropriate dose of EGF and treated with DMSO or 100 nM SB216763 (Selleckchem, Houston, TX, USA) for 3 days. Cells were harvested by centrifugation, disaggregated, and resuspended in Annexin V Binding Buffer, PE Annexin V and 7-AAD (BD Iberia, Madrid, Spain) and analyzed in a FACSCanto-A flow cytometer (BD) using FACSDiva 6.1.3 software (BD). Data were analysed with FlowJo software (Tree Star).

#### Preparation of protein extracts and immunoblot analyses

Cell extracts were obtained by lysis in SDS-buffer (25 mM Tris-HCl, pH 7.4, 1 mM EDTA, 1% SDS) or in RIPA buffer (25 mM Tris-HCl, pH 7.6, 1% Triton X-100, 0.5% sodium deoxycholate, 0.1% SDS, 150 mM NaCl) with protease and phosphatase inhibitors for 30 min at 4 °C. Lysates were centrifuged for 30 min at 14,000 rpm at 4 °C, and supernatant was considered the total lysate or total lysate soluble fraction. Lateral ventricles, including the SEZ, and whole hippocampi were dissected, frozen in dry ice and homogenized in RIPA buffer as above using potter and Polytron homogenizers. Protein concentration was determined with BCA Protein Assay kit (Pierce Biotechnology Inc., Waltham, MA, USA); 30–100 μg of protein were resolved by SDS-PAGE and transferred to nitrocellulose membranes (Protran, Sigma-Aldrich). Membranes were blocked in PBS or TBS containing 3% BSA or 5% skimmed milk, and 0.025% Tween-20 and incubated with primary antibodies (Table S2), followed by appropriate peroxidase-conjugated anti-mouse or antirabbit IgGs (Dako). Signal was obtained by enhanced chemiluminescence (Western Lightning. Perkin Elmer, Waltham, MA, USA). Autoradiographic films were scanned, and the bands were analyzed by densitometry using Image-J software (NIH). Immunoblot images have been cropped for presentation. Full-size images are presented in Supplemental Material, as Uncropped gels.

### Statistical analyses

Analyses of significant differences between means were performed with Prism 9 software (GraphPad, San Diego, CA) as specified in each figure legend. Results are shown as mean ± s.e.m. and the number of experiments (N) carried out with independent subjects (primary cultures, cells or mice) is shown in each figure as dots and is specified in the figure legends. Data were tested for normality using the Shapiro-Wilk test and homogeneity of variances was tested by F test, the Brown-Forsythe test and Bartlett’s test. Data transformations using square root, log2 or arcsin were performed to meet test assumptions when needed. Welch’s correction or a non-parametric test were used if homoscedasticity or normality were not met. Outliers were identified and removed using the ROUT method or Grubbs´test. In all cases, *P* < 0.05 denoted statistical significance. Appropriate *post-hoc* tests were applied when necessary. Investigators were not blinded to allocation during the experiments, use of animals or outcome assessment. For animal studies and experiments with animal derived tissues and cells no randomization was used and no statistical method was used to predetermine sample size. Sample sizes were based on previous published experiments.

## Supplementary information

Full and uncropped western blots

Supplementary Information

AUTHOR AGREEMENT TO THE ADDITION ON DR C LOPEZ-MENENDEZ AND A SIMON-GARCIA

reproducibility checklist

## Data Availability

All data are available under reasonable request.
