# Supplementary figures and images for: Kidins220 sets the threshold for survival of neural stem cells and progenitors to sustain adult neurogenesis
Source: Cell Death Dis. 2023 Aug 4;14(8):500. doi: 10.1038/s41419-023-05995-7 (PMC10403621; doi:10.1038/s41419-023-05995-7)

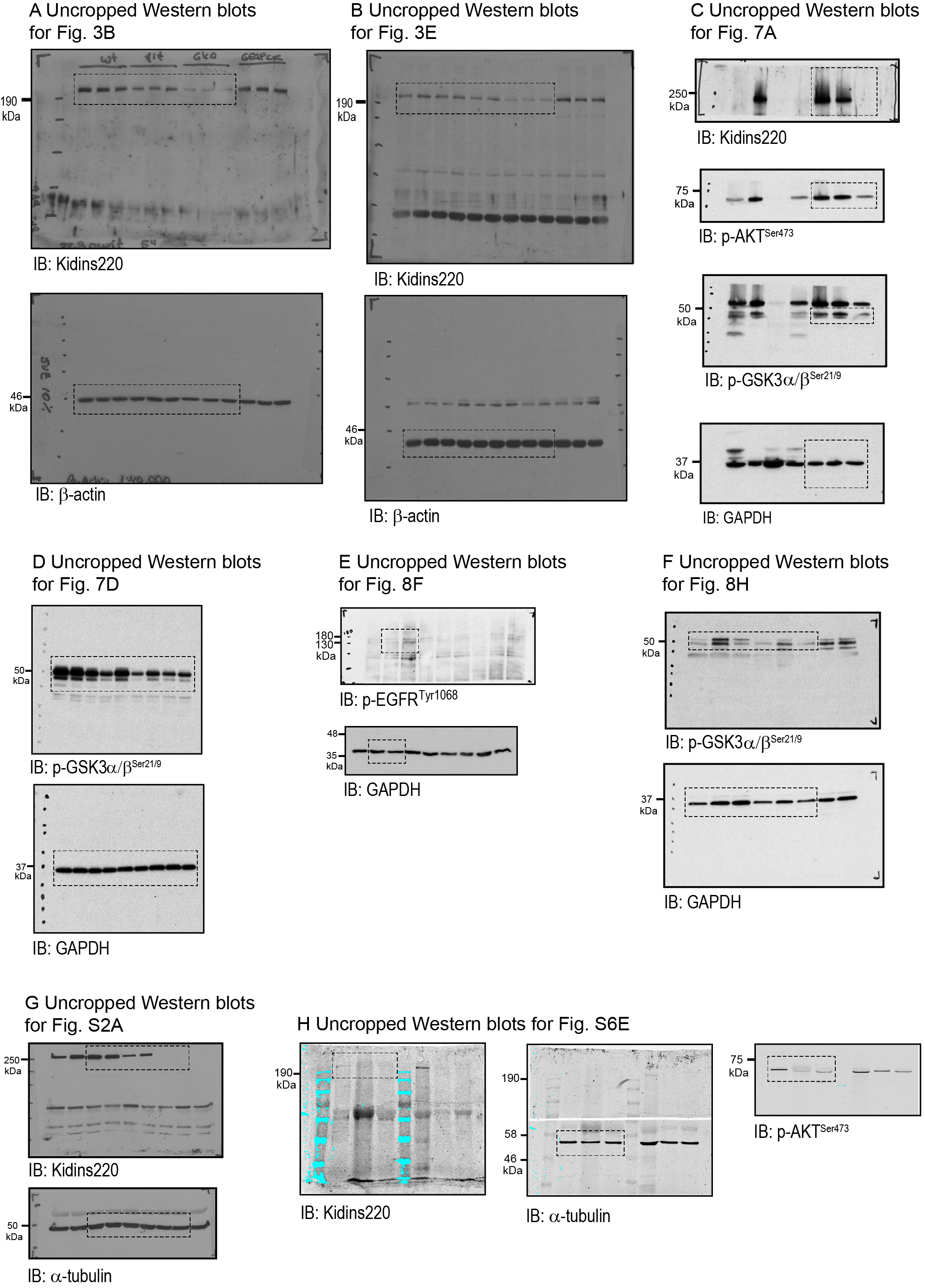

Supplement: Supplementary file 1 — Full and uncropped western blots [file 41419_2023_5995_MOESM1_ESM.tif]
