# Supplementary material for: Kidins220 sets the threshold for survival of neural stem cells and progenitors to sustain adult neurogenesis
Source: Cell Death Dis. 2023 Aug 4;14(8):500. doi: 10.1038/s41419-023-05995-7 (PMC10403621; doi:10.1038/s41419-023-05995-7)
Supplement: Supplementary file 2 — Supplementary Information [file 41419_2023_5995_MOESM2_ESM.pdf]

Kidins220 sets the threshold for survival of neural stem cells and progenitors to sustain adult neurogenesis

Ana del Puerto[1,2,§,#], Coral Lopez-Fonseca[3,4,5,#], Ana Simón-García[1,2,#], Beatriz Martí-Prado[2,6], Ana L. Barrios-Muñoz[3,4,5], Julia Pose-Utrilla[1,2,§], Celia López-Menéndez[1,2], Berta Alcover-Sanchez[3,4,5], Fabrizia Cesca[7], Giampietro Schiavo[8,9], Miguel R Campanero[5,10,11], Isabel Fariñas[2,3], Teresa Iglesias[1,2,¶,*], Eva Porlan[3,4,5,11¶,*]

[1]Instituto de Investigaciones Biomédicas "Alberto Sols". Consejo Superior de Investigaciones Científicas-Universidad Autónoma de Madrid (CSIC-UAM). C/ Arturo Duperier, 4, 28029 Madrid, Spain.
[2]Centro de Investigación Biomédica en Red de Enfermedades Neurodegenerativas (CIBERNED), Instituto de Salud Carlos III, Av. Monforte de Lemos, 3-5. Pabellón 11. Planta 0 28029 Madrid, Spain.
[3]Departamento de Biología Molecular, Universidad Autónoma de Madrid, C/ Francisco Tomás y Valiente, 7, Ciudad Universitaria de Cantoblanco, 28049 Madrid (Spain).
[4]Centro de Biología Molecular "Severo Ochoa" (CSIC-UAM), C/ Nicolás Cabrera, 1, 28049 Madrid, Spain.
[5]Instituto Universitario de Biología Molecular – UAM, C/ Nicolás Cabrera, 1, 28049 Madrid, Spain.
[6]Departmento de Biología Celular, Biología Funcional y Antropología Física, Universidad de Valencia, C/ Dr. Moliner, 50, 46100 Burjassot, Spain.
[7]Department of Life Sciences, University of Trieste, via L. Giorgieri, 5, 34127, Trieste, Italy.
[8]Department of Neuromuscular Disorders, UCL Institute of Neurology, University College London, London, WC1N 3BG, UK.
[9]UK Dementia Research Institute, University College London, London WC1E 6BT, UK.
[10]Centro de Investigación Biomédica en Red en Enfermedades Cardiovasculares (CIBERCV), Instituto de Salud Carlos III, Av. Monforte de Lemos, 3-5. Pabellón 11. Planta 0 28029 Madrid, Spain.
[11]Instituto de Investigación Sanitaria del Hospital Universitario La Paz (IdiPAZ), Instituto de Salud Carlos III, Av. Monforte de Lemos, 3-5. Pabellón 11. Planta 0 28029 Madrid, Spain.
[§]Current address: AP, Departamento de Biotecnología, Instituto Nacional de Investigación y Tecnología Agraria y Alimentaria (INIA-CSIC), Autovía A6, Km 7,5; 28040 Madrid, Spain; JP-U, Centro de Biología Molecular "Severo Ochoa" (CSIC-UAM), C/ Nicolás Cabrera, 1, 28049 Madrid, Spain.

[*]These authors contributed equally to this work.
[#]These authors contributed equally to this work
¶Correspondence and requests for materials should be addressed to Teresa Iglesias (Instituto de Investigaciones Biomédicas "Alberto Sols" (CSIC-UAM) C/ Arturo Duperier, 4 – 28029 Madrid (Spain). Phone: (+34) 915.854.487. email: tiglesias@iib.uam.es) or to Eva Porlan (Dept. de Biología Molecular y Centro de Biología Molecular "Severo Ochoa" Universidad Autónoma de Madrid. C/ Nicolás Cabrera, 1 28049 – Madrid. Phone: (+34) 911.964.629. email: eva.porlan@uam.es).

This PDF file includes:

Figures S1 to S6
Tables S1 to S3

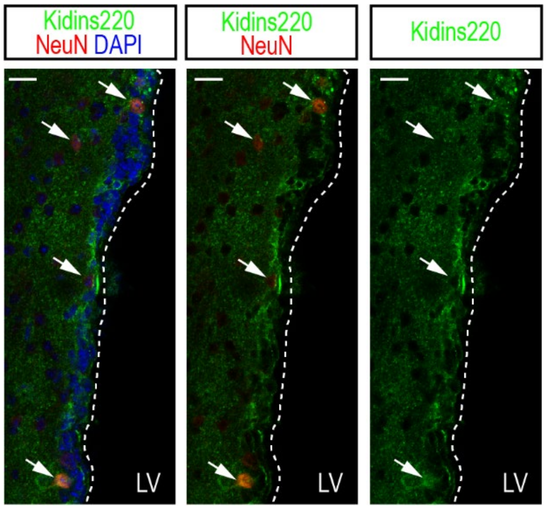

Fig. S1. Kidins220 expression in NeuN-positive neurons from the subependymal zone. Kidins220 (green) is expressed in neurons positive for NeuN (red, white arrows) in 2 month-old wild-type mice. Nuclei are stained with DAPI (blue). Scale bar, 20 μm. Dashed white lines mark the limits of the subependymal zone with the ventricle. LV, lateral ventricle.

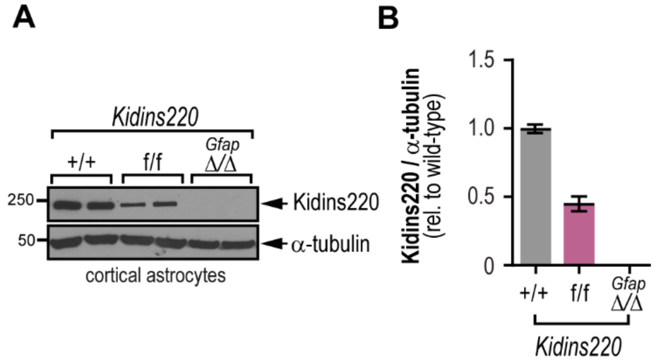

Fig. S2. Confirmation of the full excision of the floxed allele in astrocytes from *Kidins220*$^{Gfap\Delta/\Delta}$ mice. A, Immunoblot for Kidins220 and α-tubulin (loading control) in primary cortical astrocytes from the denoted genotypes. **B**, Kidins220 levels in arbitrary units after normalization with α-tubulin in wild-type and Kidins220 genetically modified mice. Data represent mean ± s.e.m. (N=2, for each genotype).

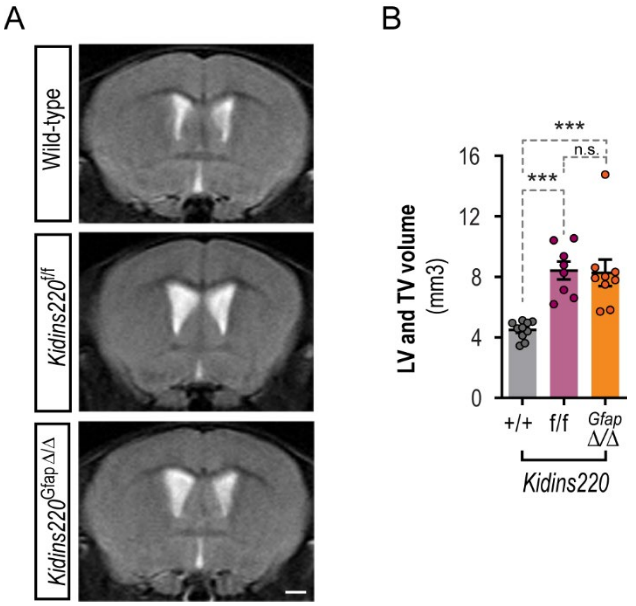

Fig. S3. *Kidins220$^{Gfap\Delta/\Delta}$* mice present ventriculomegaly. A, Representative *in vivo* T2-weighted (T2-W) MRI coronal images showing lateral and third ventricles (LVs + TV) of 2-month-old wild-type, *Kidins220$^{f/f}$* and *Kidins220$^{Gfap\Delta/\Delta}$* male mice. Scale bar, 1 mm. **B**, Quantification of LV and TV ventricular volume of 10 wild-type, 8 *Kidins220$^{f/f}$* and 9 *Kidins220$^{Gfap\Delta/\Delta}$* mice. Graph represents mean ± s.e.m. where each dot denotes values from an individual mouse (N=8-10, for each genotype). Ns, not significant, *** P < 0.001, by one-way ANOVA, followed by Tukey's *post-hoc* test.

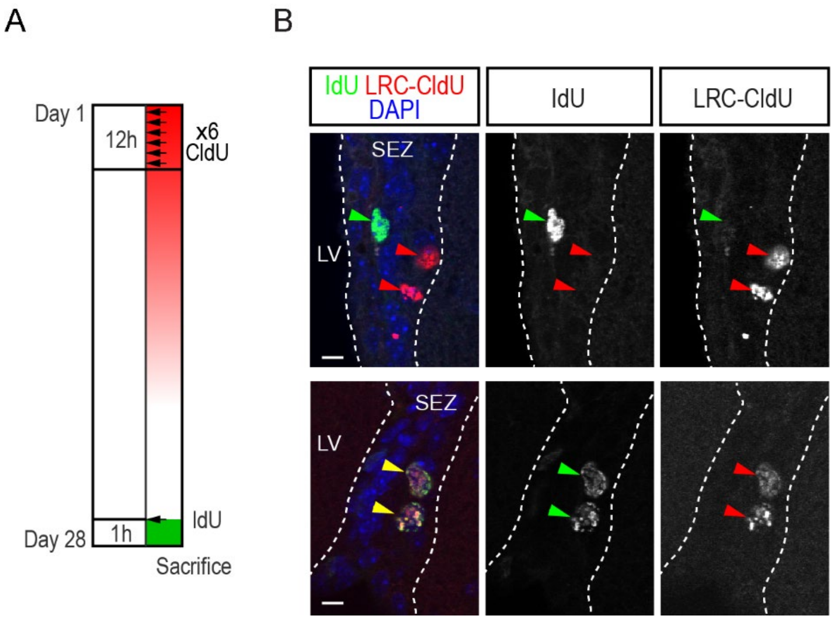

Fig. S4. CldU and IdU stainings in the murine subependymal zone. A, Schematic of the thymidine analogues regime used in this study. **B,** Representative confocal micrographs of the simultaneous staining for CldU (red) and IdU (green) in the subependymal zone (comprised between the white dashed lines) of 2-month-old wild-type mice. Green and red arrowheads point to single-labeled IdU⁺ and CldU⁺ cells, respectively. Yellow arrowheads point at double-labelled cells, in the merged image. Scale bar, 5 μm. LV, lateral ventricle.

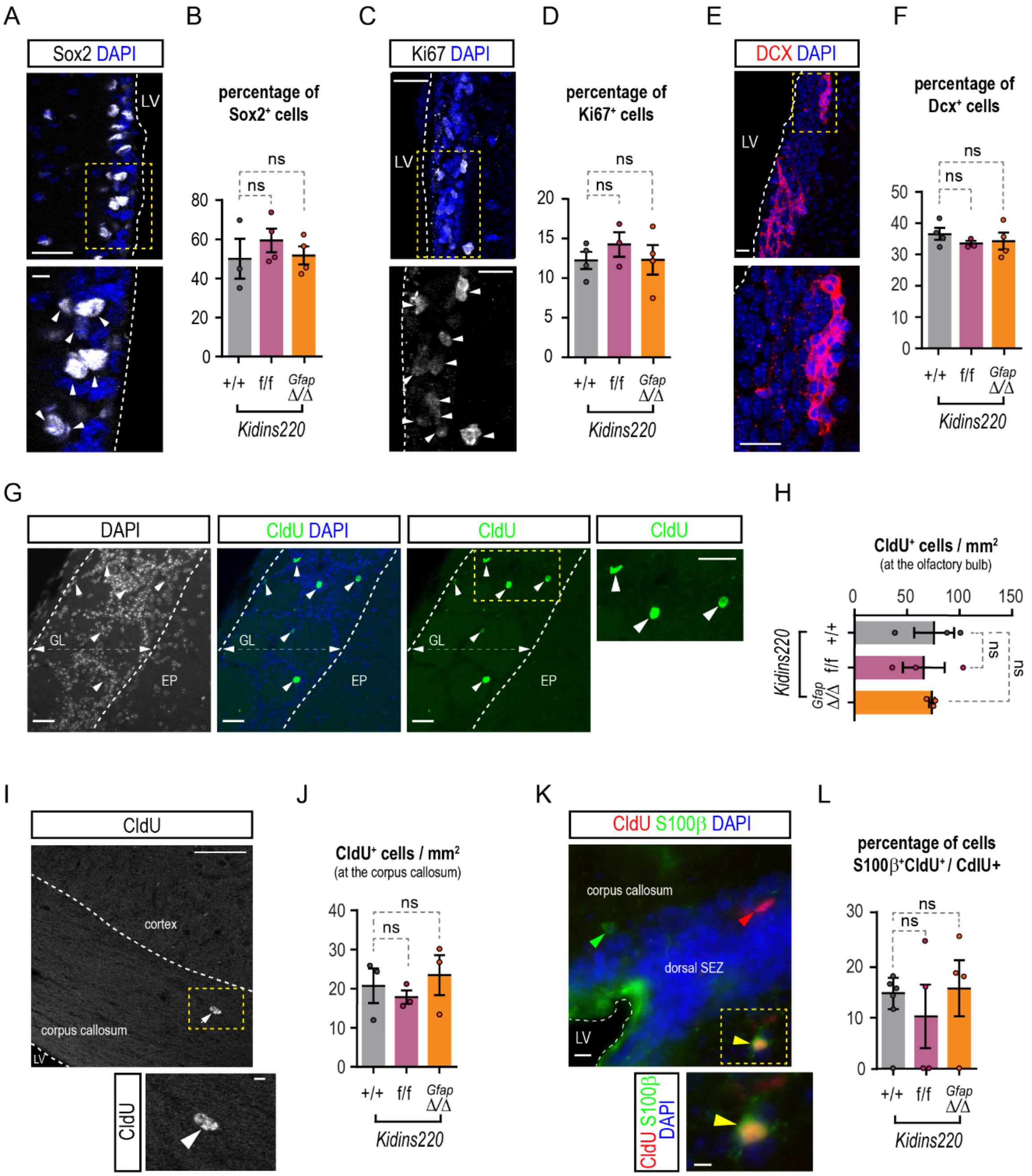

Fig. S5. Adult cytogenesis from the 2 month-old SEZ and *corpus callosum* is Kidins220 independent. Representative confocal micrographs and quantifications of the percentage of Sox2$^+$ (gray, white arrowheads, **A, B**), Ki67$^+$ (gray, white arrowheads, **C, D**) and Doublecortin (DCX)$^+$ (red, **E, F**) in the subependymal zone of 2 month-old wild-type and Kidins220 genetically modified mice. Nuclei are stained with DAPI (blue). Sox2$^+$-ependymal cells were excluded from the analysis. Mice were injected with CldU (green) as described in methods section and sacrificed 28 days later. CldU$^+$

cells (white arrowheads) in the glomerular layer of the OBs (**G, H**) and in the *corpus callosum* (**I, J**) were scored. **K**, Representative confocal micrographs of CldU$^+$ cells (red), double positive (yellow arrowhead) for the astrocytic marker S100-β (green, green arrowhead) were quantified in the SEZ of wild-type and Kidins220 genetic models (**L**). Graphs represent mean ± s.e.m. where each data point denotes an individual mouse (N=3-5, for each genotype). Ns, not significant. One-way ANOVA, followed by Dunnett's *post-hoc* test. White dashed lines mark the boundary with the ventricle lumen (A, C, E and K). GL, granular layer (enclosed within the white dashed lines, G), EP, external plexiform layer. White dashed lines mark the limit with the lateral ventricle (LV) and the cortex, in **I**. Scale bars, 25 μm (A, C, E, G, K low magnification images), 50 μm (**I**, low magnification images) and 5, 10, 25, 25, 5 and 10 μm for insets (yellow dashed lines) in A, C, E, G, **I** and K, respectively.

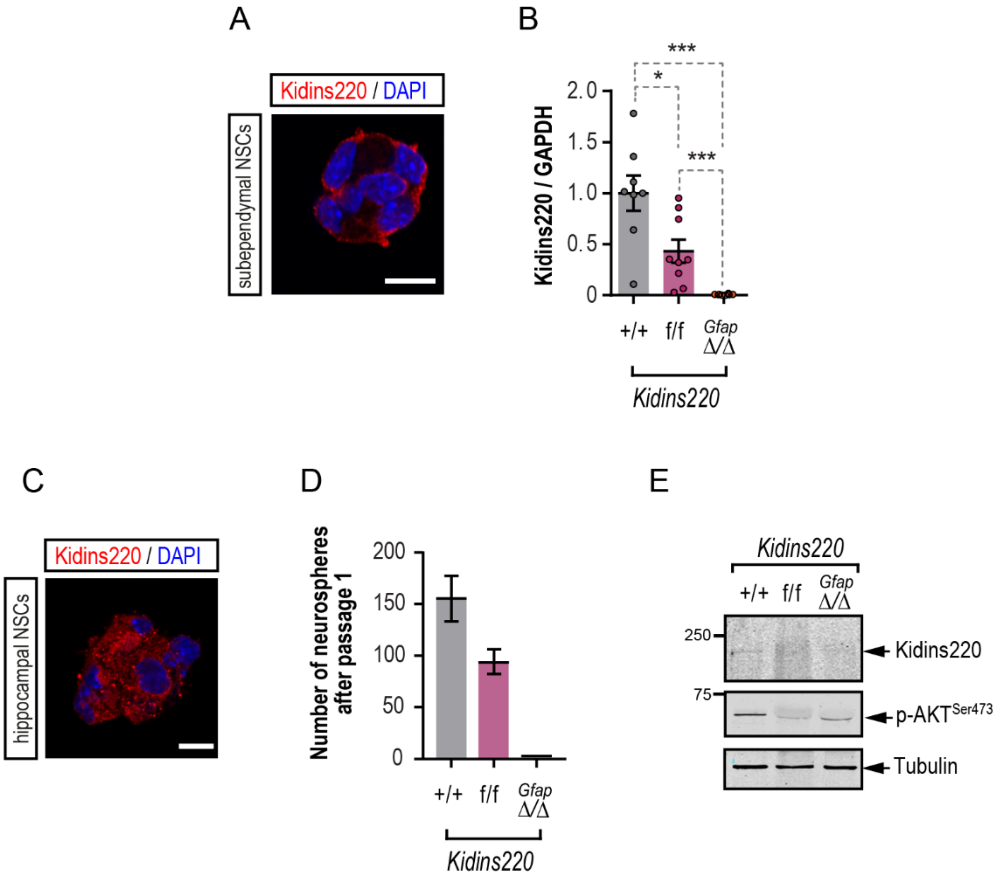

Fig. S6. Kidins220 expression in neurospheres from wild-type and Kidins220 genetic models. A, **C**, Kidins220 (red) is highly expressed in SEZ and hippocampal-derived neurospheres. Nuclei are stained with DAPI (blue). Scale bar, 10 μm. **B,** Kidins220 levels represented in arbitrary units after normalization with GAPDH and relative to wild-type in extracts from wild-type, *Kidins220*$^{f/f}$ and *Kidins220*$^{Gfap\Delta/\Delta}$ mice after immunoblot analysis of lysates from neurospheres obtained from lateral ventricle walls. Data represent mean ± s.e.m. Each data point represents a neurosphere culture established from an independent mouse (N=8-9, for each genotype). *$P < 0.05$, *** $P < 0.0001$ by one-way ANOVA followed by Tukey's *post-hoc* test. **D**, Number of hippocampal neurospheres obtained after the first passage from wild-type (N=2), *Kidins220*$^{f/f}$ (N=3) and *Kidins220*$^{Gfap\Delta/\Delta}$ (N=4) mice. No neurospheres grew from *Kidins220*$^{Gfap\Delta/\Delta}$ mice. **E**, Immunoblot for Kidins220, p-AKT^{Ser473} and α-tubulin (loading control) in primary hippocampal neurospheres from the denoted genotypes.

Table S1. Sequences of oligonucleotides used for genotyping

Sequence	*Use*
5′- GAGCACAGACTTCTCTTATGG -3′	Forward primer for detection of *Kidins220*-floxed and recombined alleles
5′- GCGTTTCTAGCATACACATG -3′	Reverse primer for detection of the *Kidins220* floxed allele
5′-CAGATGGCTGTGAACCACCGTTTAAAC-3′	Reverse primer for detection of the *Kidins220* recombined allele
5′- GCGGTCTGGCAGTAAAAACTATC -3′	Forward primer for detection of *Cre*
5′- GTGAAACAGCATTGCTGTCACTT -3′	Reverse primer for detection of *Cre*
5′- CTAGGCCACAGAATTGAAAGATCT -3′	Forward primer for detection of internal control DNA
5′- GTAGGTGGAAATTCTAGCATCATCC -3′	Reverse primer for detection of internal control DNA

Table S2. Primary antibodies used in this work

Antigen (Species)	*Working dilution & use*	*Origin*
Kidins220 (Rb)	1:500 IF/IHC	(15) from T. Iglesias lab.
Kidins220 (Rb)	1:250 IF/IHC	(10) from T. Iglesias lab.
Kidins220 (Rb)	1:1000 IB	(10) from T. Iglesias lab.
Sox2 (Rb)	1:300 IHC	Abcam ab97959
Sox2 (Gt)	1:250 IHC	R&D Systems AF2018
Ki67 (Rb)	1:200 IF	Abcam ab15580
IdU (M)	1:500 IHC	BD 347580
CldU (R)	1:500 IHC	Abcam ab6326
GFAP (Ch)	1:500 IHC	Sigma-Aldrich AB5541
γ-Tubulin (M)	1:500 IHC	Sigma-Aldrich T6557
γ-Tubulin (Rb)	1:250 IHC	Abcam ab11317
S100-β (Rb)	1:100 IHC	Abcam ab52642
GAPDH (M)	1:1000 IB	Sigma-Aldrich MAB374
α-Tubulin (M)	1:10 000 IB	Sigma-Aldrich T9026
β-Actin (M)	1:40 000 IB	Sigma-Aldrich A2228
p-GSK3α/β$^{S21/9}$ (Rb)	1.1000 IB	Cell Signaling Technology #9331
p-AKT^{S473} (Rb)	1:1000 IB	Cell Signaling Technology #4060
AKT (M)	1:1000 IB	Cell Signaling Technology #2966S
N-Cadherin (M)	1:100 IHC; 1:1000 IF	BD Transduction Laboratories AB_398236
β-Catenin (Rb)	1:100 IHC	Cell Signaling Technology #9562
Cleaved caspase-3 (Rb)	1:250 IF	Cell Signaling Technology #9661
NeuN (M)	1:2000 IF	Sigma-Aldrich MAB377
Doublecortin (Dcx) (Gt)	1:250 IF	Santa Cruz Biotechnology sc-8066
p-EGFR^{Y1068} (Rb)	1:250 IB; 1:300 IF	Cell Signaling Technology #2234
EGFR (M)	1:1000 IB; 1:500 IF	Santa Cruz Biotechnology sc-373746
Mash1 (Ascl1) (M)	1:100 IF	BD Pharmingen 556604

IF, immunofluorescence; IHC, immunohistochemistry; IB, immunoblot; Rb, rabbit; M, mouse; Gt, goat; R, rat; Ch, chicken.

Table S3. Two-way RM ANOVA related to Fig. 3A

		Interaction	Genotype	Session
**Acquisition Phase**	**Latency**	F (8, 104) = 0.9036<br>P=0.5165 ns	F (3.269, 85.00) = 80.70<br>P<0.0001 ***	F (2, 26) = 1.002<br>P=0.3807 ns
	**Accuracy**	F (8, 104) = 1.290<br>P=0.2566 ns	F (3.385, 88.01) = 41.61<br>P<0.0001 ***	F (2, 26) = 0.2263<br>P=0.7990 ns
	**Wrong Visits**	F (8, 104) = 1.160<br>P=0.3305 ns	F (3.055, 79.43) = 39.12<br>P<0.0001 ***	F (2, 26) = 0.5582<br>P=0.5790 ns
**Reversal phase**	**Latency**	F (8, 104) = 0.8745<br>P=0.5405 ns	F (2.178, 56.63) = 5.403<br>P=0.0058 **	F (2, 26) = 10.46<br>P=0.0005 ***
	**Accuracy**	F (8, 104) = 0.8048<br>P=0.5997 ns	F (3.223, 83.79) = 9.367<br>P<0.0001 ***	F (2, 26) = 16.66<br>P<0.0001 ***
	**Wrong Visits**	F (8, 104) = 1.006<br>P=0.4360 ns	F (2.317, 60.24) = 5.948<br>P=0.0029 **	F (2, 26) = 5.953<br>P=0.0074 **
