# Supplementary material for: Kidins220 sets the threshold for survival of neural stem cells and progenitors to sustain adult neurogenesis
Source: Cell Death Dis. 2023 Aug 4;14(8):500. doi: 10.1038/s41419-023-05995-7 (PMC10403621; doi:10.1038/s41419-023-05995-7)
Supplement: Supplementary file 3 — AUTHOR AGREEMENT TO THE ADDITION ON DR C LOPEZ-MENENDEZ AND A SIMON-GARCIA [file 41419_2023_5995_MOESM3_ESM.pdf]

Dear Sir/Madam,

As per instructions provided by the Editorial Office, I have requested agreement from all authors to the new additions in the author list. We have included the agreement from the newly added authors as well just in case this was also necessary.

I have collected their replies by email and have generated a combined PDF document that follows my own agreement statement (below).

I, EVA PORLAN, *agree to the addition of Ana Simón-García and Dr. Celia López-Menéndez to the list of authors of the manuscript CDDIS-22-3643RR.*

Sincerely,

Eva Porlan

--
Eva Porlan, PhD
Associate Professor
Group Leader Neural Stem Cells in the Adult Brain
Dept. de Biología Molecular y Centro de Biología Molecular "Severo Ochoa"
Universidad Autónoma de Madrid
C/ Nicolás Cabrera, 1 28049 - Madrid
eva.porlan@cbm.uam.es
eva.porlan@uam.es
Tel: +34 911 964 629

Asunto: Re: CDDis quality check
De: Teresa Iglesias Vacas <tiglesias@iib.uam.es>
Fecha: 05/07/2023, 18:15
Para: Eva Porlan <eva.porlan@uam.es>, Beatriz Martí <beatriz.marti.prado@gmail.com>, Ana Laura Barrios <analaurabarrios@yahoo.es>, Coral Lopez Fonseca <coral.lopez@cbm.csic.es>, Julia Pose Utrilla <jpose@ext.iib.uam.es>, berta.alcover@cbm.csic.es, giampietro.schiavo@ucl.ac.uk, fcesca@units.it, Isabel Fariñas <isabel.farinas@uv.es>, mcampanero@cbm.csic.es, clopez@iib.uam.es, Ana Simón García <asimon@iib.uam.es>, bmarti1@terraroja.cat

I, TERESA IGLESIAS agree to the addition of Ana Simón-García and Dr. Celia López-Menéndez to the list of authors of the manuscript CDDIS-22-3643RR.

--
Teresa Iglesias Vacas, Ph.D.
Group Leader
Novel Targets in Neurodegeneration and Neuroprotection Lab
Endocrine and Nervous System Pathophysiology Department
Instituto de Investigaciones Biomédicas "Alberto Sols" (CSIC-UAM)
and CIBERNED (ISCIII)
C/ Arturo Duperier, 4 – 28029 Madrid (Spain)
Tel: (+34) 91.585.44.87
email: tiglesias@iib.uam.es
____

Asunto: Fwd: CDDis quality check
De: Ana M del Puerto <delpuerto.ana@inia.csic.es>
Fecha: 05/07/2023, 19:05
Para: <eva.porlan@cbm.uam.es>
CC: <tiglesias@iib.uam.es>

I, <u>Ana del Puerto</u>, agree to the addition of Ana Simón-García and Dr. Celia López-Menéndez to the list of authors of the manuscript CDDIS-22-3643RR.

Asunto: Re: CDDis quality check
Fecha: Wed, 05 Jul 2023 16:56:06 +0200
De: CORAL LOPEZ FONSECA <coral.lopez@cbm.csic.es>
Para: Eva Porlan <eva.porlan@uam.es>
CC: Teresa Iglesias Vacas <tiglesias@iib.uam.es>

Dear Teresa and Eva,

Since the author list has changed with the addition of Ana Simón-García and Celia López-Menéndez, I confirm below that I am in agreement with these changes:

I, Coral Lopez-Fonseca, agree to the addition of Ana Simón-García and Dr. Celia López-Menéndez to the list of authors of the manuscript CDDIS-22-3643RR.

Best,
——
Coral Lopez Fonseca
Estudiante de Doctorado (PhD Candidate)
Procesos Fisiologicos y Patologicos - Neuropatologia Molecular
Centro de Biologia Molecular Severo Ochoa (Laboratorio 306)
Universidad Autonoma de Madrid • Campus de Cantoblanco
c/ Nicolas Cabrera, 1 - 28049 Madrid
Tel.: +34 91 196 46 55 - coral.lopez@cbm.csic.es

Asunto: Re: CDDis quality check
De: Ana Simón García <asimon@iib.uam.es>
Fecha: 05/07/2023, 17:05
Para: Eva Porlan <eva.porlan@uam.es>
CC: Teresa Iglesias Vacas <tiglesias@iib.uam.es>

I, Ana Simón García agree to the addition of Ana Simón-García and Dr. Celia López-Menéndez to the list of authors of the manuscript CDDIS-22-3643RR.

Ana Simón García
Ph.D. Student
Novel Targets in Neurodegeneration and Neuroprotection Lab
Endocrine and Nervous System Pathophysiology Department
Instituto de Investigaciones Biomédicas Alberto Sols" (CSIC-UAM)
and CIBERNED (ISCIII)
C/ Arturo Duperier, 4
Madrid 28029
Spain

Tel.: +34 915854488
E-mail: asimon@iib.uam.es

Asunto: Re: CDDis quality check
De: Beatriz MARTÍ <bmarti1@terraroja.cat>
Fecha: 05/07/2023, 19:34
Para: CESCA FABRIZIA <fcesca@units.it>
CC: Teresa Iglesias Vacas <tiglesias@iib.uam.es>, Eva Porlan <eva.porlan@uam.es>, Beatriz Martí <beatriz.marti.prado@gmail.com>, Ana Laura Barrios <analaurabarrios@yahoo.es>, Coral Lopez Fonseca <coral.lopez@cbm.csic.es>, Julia Pose Utrilla <jpose@ext.iib.uam.es>, "berta.alcover@cbm.csic.es" <berta.alcover@cbm.csic.es>, "giampietro.schiavo@ucl.ac.uk" <giampietro.schiavo@ucl.ac.uk>, Isabel Fariñas <isabel.farinas@uv.es>, "mcampanero@cbm.csic.es" <mcampanero@cbm.csic.es>, "clopez@iib.uam.es" <clopez@iib.uam.es>, Ana Simón García <asimon@iib.uam.es>

I, BEATRIZ MARTÍ PRADO agree to the addition of Ana Simón-García and Dr. Celia López-Menéndez to the list of authors of the manuscript CDDIS-22-3643RR.

--

Beatriz Marí
Tutora de 1r ESO - B
Departament d'experimentals
Institut Terra Roja

De: Ana Laura Barrios <analaurabarrios@yahoo.es>
Fecha: 06/07/2023, 20:06
Para: Eva Porlan <eva.porlan@uam.es>, BERTA ALCOVER SANCHEZ <berta.alcover@cbm.csic.es>
CC: Beatriz Martí <beatriz.marti.prado@gmail.com>, Coral Lopez Fonseca <coral.lopez@cbm.csic.es>, Julia Pose Utrilla <jpose@iib.uam.es>, "giampietro.schiavo@ucl.ac.uk" <giampietro.schiavo@ucl.ac.uk>, "fcesca@units.it" <fcesca@units.it>, Isabel Fariñas <isabel.farinas@uv.es>, "mcampanero@cbm.csic.es" <mcampanero@cbm.csic.es>, "clopez@iib.uam.es" <clopez@iib.uam.es>, Ana Simón García <asimon@iib.uam.es>, "bmarti1@terraroja.cat" <bmarti1@terraroja.cat>, Teresa Iglesias <tiglesias@iib.uam.es>

I Ana Barrios-Muñoz agree to the addition of Ana Simón-García and Dr. Celia López-Menéndez to the list of authors of the manuscript CDDIS-22-3643RR.*

Asunto: I agree
De: JULIA POSE UTRILLA <jpose@cbm.csic.es>
Fecha: 05/07/2023, 19:07
Para: eva.porlan@uam.es
CC: tiglesias@iib.uam.es

I, Julia Pose-Utrilla agree to the addition of Ana Simón-García and Dr. Celia López-Menéndez to the list of authors of the manuscript CDDIS-22-3643RR.

Asunto: Re: CDDis quality check
De: Celia López Menéndez <clopez@iib.uam.es>
Fecha: 07/07/2023, 12:29
Para: Isabel.Farinas@uv.es
CC: Beatriz_Martí <beatriz.marti.prado@gmail.com>, Ana Laura Barrios <analaurabarrios@yahoo.es>, Coral Lopez Fonseca <coral.lopez@cbm.csic.es>, berta.alcover@cbm.csic.es, giampietro.schiavo@ucl.ac.uk, fcesca@units.it, mcampanero@cbm.csic.es, Ana Simón García <asimon@iib.uam.es>, bmarti1@terraroja.cat, Eva Porlan <eva.porlan@uam.es>, Teresa Iglesias <tiglesias@iib.uam.es>

I, Celia López-Menéndez agree to the addition of Ana Simón-García and Dr. Celia López-Menéndez to the list of authors of the manuscript CDDIS-22-3643RR.

Best, Celia

Asunto: Re: CDDis quality check
De: BERTA ALCOVER SANCHEZ <berta.alcover@cbm.csic.es>
Fecha: 06/07/2023, 8:57
Para: Eva Porlan <eva.porlan@uam.es>
CC: Beatriz Martí <beatriz.marti.prado@gmail.com>, Ana Laura Barrios <analaurabarrios@yahoo.es>, Coral Lopez Fonseca <coral.lopez@cbm.csic.es>, Julia Pose Utrilla <jpose@iib.uam.es>, giampietro.schiavo@ucl.ac.uk, fcesca@units.it, Isabel Fariñas <isabel.farinas@uv.es>, mcampanero@cbm.csic.es, clopez@iib.uam.es, Ana Simón García <asimon@iib.uam.es>, bmarti1@terraroja.cat, Teresa Iglesias <tiglesias@iib.uam.es>

*I, **BERTA ALCOVER-SANCHEZ** agree to the addition of Ana Simón-García and Dr. Celia López-Menéndez to the list of authors of the manuscript CDDIS-22-3643RR.*

Berta Alcover Sánchez
PhD Student in Molecular Biosciences
Personal de Investigación
Neuropatología Molecular
Centro de Biología Molecular Severo Ochoa - Laboratorio 305
C/ Nicolás Cabrera, 1 - 28049 Madrid
Tel.: +34 91 196 4650 - berta.alcover@cbm.csic.es

Asunto: Re: CDDis quality check
De: CESCA FABRIZIA <fcesca@units.it>
Fecha: 05/07/2023, 18:40
Para: Teresa Iglesias Vacas <tiglesias@iib.uam.es>
CC: Eva Porlan <eva.porlan@uam.es>, Beatriz Martí <beatriz.marti.prado@gmail.com>, Ana Laura Barrios <analaurabarrios@yahoo.es>, Coral Lopez Fonseca <coral.lopez@cbm.csic.es>, Julia Pose Utrilla <jpose@ext.iib.uam.es>, "berta.alcover@cbm.csic.es" <berta.alcover@cbm.csic.es>, "giampietro.schiavo@ucl.ac.uk" <giampietro.schiavo@ucl.ac.uk>, Isabel Fariñas <isabel.farinas@uv.es>, "mcampanero@cbm.csic.es" <mcampanero@cbm.csic.es>, "clopez@iib.uam.es" <clopez@iib.uam.es>, Ana Simón García <asimon@iib.uam.es>, "bmarti1@terraroja.cat" <bmarti1@terraroja.cat>

I, FABRIZIA CESCA agree to the addition of Ana Simón-García and Dr. Celia López-Menéndez to the list of authors of the manuscript CDDIS-22-3643RR.

Fabrizia Cesca, PhD
Prof.ssa Associata | Associate Professor
Delegata DSV per la mobilità internazionale | Dept. Delegate for Int. Mobility
Dipartimento di Scienze della Vita | Department of Life Sciences
Università degli Studi di Trieste | University of Trieste
Building Q, room 213 - via L. Giorgieri, 5 - 34127 Trieste (Italy)
fcesca@units.it
Tel. | Ph. +39 040 558 8727
Cell. | Mob. +39 345 3598007
Skype: fabriziacesca

Asunto: Re: CDDis quality check
De: "Schiavo, Giampietro" <giampietro.schiavo@ucl.ac.uk>
Fecha: 05/07/2023, 17:07
Para: Eva Porlan <eva.porlan@uam.es>

Dear Sir/Madam,

I, Giampietro Schiavo agree to the addi�on of Ana Simón-García and Dr. Celia López-Menéndez to the list of authors of the manuscript CDDIS-22-3643RR.

Best regards
gipi

Asunto: Re: CDDis quality check
De: Miguel Campanero <mcampanero@cbm.csic.es>
Fecha: 05/07/2023, 17:07
Para: Eva Porlan <eva.porlan@uam.es>, Teresa_Iglesias <tiglesias@iib.uam.es>

Dear Eva and Teresa,

I, Dr. Miguel R. Campanero, agree to the addition of Ana Simón-Garcia and Dr. Celia López-Menéndez to the list of authors of the manuscript CDDIS-22-3643RR.

Best regards,
Miguel
--
Miguel R. Campanero, PhD
Tenured Scientist and Group Leader
AECC Researcher
Centro de Biología Molecular Severo Ochoa
CSIC-UAM
Nicolás Cabrera, 1
28049 Madrid
Spain
Tel: +34-91-196-4554
Email: mcampanero@cbm.csic.es
WEB: http://www.cbm.uam.es/mcampanero

Asunto: Re: CDDis quality check
De: <Isabel.Farinas@uv.es>
Fecha: 06/07/2023, 22:32
Para: "Beatriz_Martí" <beatriz.marti.prado@gmail.com>, "Ana Laura Barrios" <analaurabarrios@yahoo.es>, "Coral Lopez Fonseca" <coral.lopez@cbm.csic.es>, "Julia Pose Utrilla" <jpose@iib.uam.es>, berta.alcover@cbm.csic.es, giampietro.schiavo@ucl.ac.uk, fcesca@units.it, "Isabel_Fariñas" <isabel.farinas@uv.es>, mcampanero@cbm.csic.es, clopez@iib.uam.es, "Ana Simón García" <asimon@iib.uam.es>, bmarti1@terraroja.cat, "Eva Porlan" <eva.porlan@uam.es>, "Teresa Iglesias" <tiglesias@iib.uam.es>

I, Isabel Fariñas agree to the addition of Ana Simón-García and Dr. Celia López-Menéndez to the list of authors of the manuscript CDDIS-22-3643RR.

Best, Isabel

--
****
Isabel Fariñas
Biología Celular

Universidad de Valencia
46100 Burjassot
Spain
Phone: 34-963 543784 (office) 3246 (lab)
FAX: 34-963 544372
****
